# A network-based transcriptomic landscape of HepG2 cells uncovering causal gene-cytotoxicity interactions underlying drug-induced liver injury

**DOI:** 10.1093/toxsci/kfad121

**Published:** 2023-11-28

**Authors:** Lukas S Wijaya, Attila Gabor, Iris E Pot, Luca van de Have, Julio Saez-Rodriguez, James L Stevens, Sylvia E Le Dévédec, Giulia Callegaro, Bob van de Water

**Affiliations:** Leiden Academic Centre for Drug Research (LACDR), Faculty of Science, Leiden University, 2333 Leiden, The Netherlands; Institute for Computational Biomedicine, Faculty of Medicine, Heidelberg University, 69120 Heidelberg, Germany; Heidelberg University Hospital, Molecular Medicine Partnership Unit, 69120 Heidelberg, Germany; Leiden Academic Centre for Drug Research (LACDR), Faculty of Science, Leiden University, 2333 Leiden, The Netherlands; Leiden Academic Centre for Drug Research (LACDR), Faculty of Science, Leiden University, 2333 Leiden, The Netherlands; Institute for Computational Biomedicine, Faculty of Medicine, Heidelberg University, 69120 Heidelberg, Germany; Heidelberg University Hospital, Molecular Medicine Partnership Unit, 69120 Heidelberg, Germany; Leiden Academic Centre for Drug Research (LACDR), Faculty of Science, Leiden University, 2333 Leiden, The Netherlands; Leiden Academic Centre for Drug Research (LACDR), Faculty of Science, Leiden University, 2333 Leiden, The Netherlands; Leiden Academic Centre for Drug Research (LACDR), Faculty of Science, Leiden University, 2333 Leiden, The Netherlands; Leiden Academic Centre for Drug Research (LACDR), Faculty of Science, Leiden University, 2333 Leiden, The Netherlands

**Keywords:** hepatotoxicity, toxicogenomics, high throughput, cytotoxicity, biomarkers, WGCNA

## Abstract

Drug-induced liver injury (DILI) remains the main reason for drug development attritions largely due to poor mechanistic understanding. Toxicogenomic to interrogate the mechanism of DILI has been broadly performed. Gene coregulation network-based transcriptome analysis is a bioinformatics approach that potentially contributes to improve mechanistic interpretation of toxicogenomic data. Here we performed an extensive concentration time course response-toxicogenomic study in the HepG2 cell line exposed to 20 DILI compounds, 7 reference compounds for stress response pathways, and 10 agonists for cytokines and growth factor receptors. We performed whole transcriptome targeted RNA sequencing to more than 500 conditions and applied weighted gene coregulated network analysis to the transcriptomics data followed by the identification of gene coregulated networks (modules) that were strongly modulated upon the exposure of DILI compounds. Preservation analysis on the module responses of HepG2 and PHH demonstrated highly preserved adaptive stress response gene coregulated networks. We correlated gene coregulated networks with cell death onset and causal relationships of 67 critical target genes of these modules with the onset of cell death was evaluated using RNA interference screening. We identified *GTPBP2*, *HSPA1B*, *IRF1*, *SIRT1*, and *TSC22D3* as essential modulators of DILI compound-induced cell death. These genes were also induced by DILI compounds in PHH. Altogether, we demonstrate the application of large transcriptome datasets combined with network-based analysis and biological validation to uncover the candidate determinants of DILI.

Drug-induced liver injury (DILI) remains a worldwide health problem, representing approximately 20% of adverse drug reactions (David and Hamilton, 2010). Additionally, DILI is also the main cause for drug attritions in both preclinical and clinical phases (Babai *et al.*, 2021). Multiple studies have been conducted to improve the mechanistic understanding of DILI. A recent review reported multiple cellular responses that could contribute to clinical manifestation of DILI: mitochondrial impairment, inhibition of biliary efflux, lysosomal impairment, reactive metabolites, endoplasmic reticulum stress, and immune system activation (Weaver *et al.*, 2020). Despite recent advances, a more holistic understanding of the cellular events underpinning DILI outcomes and their causal relationship with adverse events starting from cell to more complex tissue and organ responses in an AOP framework is yet to be established. Currently, there is a lack of sufficient data that underpins the causality between the cellular responses and cellular outcomes in DILI episodes.

Transcriptomic approaches are promising tools to achieve a detailed description of the biological mechanisms that contribute to DILI as well as the prediction of its occurrence (Kohonen *et al.*, 2017; Li *et al.*, 2020). For example, an extensive *in vitro* study has been performed in HepaRG cell lines exposed to >1000 chemicals from the ToxCast library assessing the expression of almost 100 different genes. Despite this small gene set, this study identified transcriptomics signatures related to molecular initiating events of these compounds (Franzosa *et al.*, 2021). In other contexts, transcriptomic profiling has been reported to have higher predictability toward cellular outcomes compared with a sole interpretation from the activation of transcription factors (Weinreb *et al.*, 2020). However, interpreting transcriptomic data can be challenging due to high dimensionality of the data resulting to low signal-to-noise and overall variability (Lozoya *et al.*, 2018). Enrichment approaches such as pathway enrichment analysis and network-based analysis enable to reduce the dimensionality of transcriptomic datasets (O’Brien *et al.*, 2006). The network-based analysis is a promising approach to study complex biological responses and improve the understanding of disease pathways and the identification of potential drug targets and biomarkers (Barabási *et al.*, 2011). Such approaches could be applied to DILI-related toxicogenomic data to advance the mechanistic understanding of DILI.

Weighted gene coregulated network analysis (WGCNA) is one method to perform network-based analysis (Langfelder and Horvath, 2008). This method clusters genes based on their coexpression into specific networks called modules (gene coregulated networks—referred as gene networks in this study). Each module is scored (eigengene [EG] scores) based on the expression values of the composing genes, thus indicating an overall value of the module activity (induction or repression). In order to increase the biological meaning of the modules, each module can be linked to annotations that represent cell biological processes, signaling pathways and responses. To date, only few studies have applied WGCNA to toxicogenomic datasets. Sutherland *et al.* derived WGCNA modules from the *in vivo* transcriptomic data in rats based on the Drug Matrix dataset and demonstrated that modules facilitated to unravel mechanisms of DILI in rats and to define molecular processes that underlie preclinical outcomes, such as histopathology and clinical chemistry, which has recently been extended to identification of translational biomarkers (Callegaro *et al.*, 2023; Sutherland *et al.*, 2018). Callegaro *et al.* developed a WGCNA approach using cultured primary human hepatocytes (PHH) from the TG-GATEs dataset. WGCNA modules were able to capture the hepatocellular events related to DILI and furthermore identified the cellular events that were conserved across species (Callegaro *et al.*, 2021). So far, an attempt to systematically define the causality between gene network perturbation and onset of toxicity is lacking. Although PHH represent the gold standard for *in vitro* DILI testing, PHH have inherent limitations for genetic manipulation to uncover mechanisms. In contrast, the hepatocarcinoma HepG2 cell line, which is widely used for early DILI screening and showed relatively close resemblance to cytotoxicity responses of PHH (Sison-Young *et al.*, 2017), allows mechanistic functional genomics studies. However, so far large toxicogenomic datasets of HepG2 that allow WGCNA application are missing. Moreover, the knowledge on the preserved cellular responses linked to progressive cellular outcomes between PHH and HepG2 is undefined. The utilization of robust, cost effective, and fast liver test systems for WGCNA application will facilitate the experimental validation of causal relationships between module activation and adverse outcomes in the context of DILI.

In this study, we established a comprehensive toxicogenomic dataset with the HepG2 cell line based on exposure to more than 500 different treatment conditions. This involved established DILI compounds, selective compounds that activate specific stress response pathways, cytokines and growth factors relevant in liver injury and regeneration, and negative reference control compounds without DILI liability (Wijaya *et al.*, 2021). Cells were treated with these substances with up to 6 different concentrations and high-throughput whole-genome TempO-Seq-targeted RNA sequencing was performed at 4, 8, and 24 h. We utilized the WGCNA approach to capture the cellular events in the context of DILI compound treatment and mapped the highly perturbed cellular responses upon exposure of the DILI compounds. We performed preservation analysis with PHH and defined modules that are associated with DILI compound-induced cell death. Finally, we validated the causality between the modules that showed a strong association with DILI drug-induced cell death. From the 67 highly upregulated genes inside correlated modules, we found *GTPBP2*, *HSPA1B*, *IRF1*, *SIRT1*, and *TSC22D3* to impact on cell death onset (Figure 1A). Altogether, we established a novel quantitative network-based approach for the assessment of HepG2 toxicogenomic data that enables improved mechanistic understanding of mode-of-action of DILI compounds and novel drug candidates.

**Figure 1.**
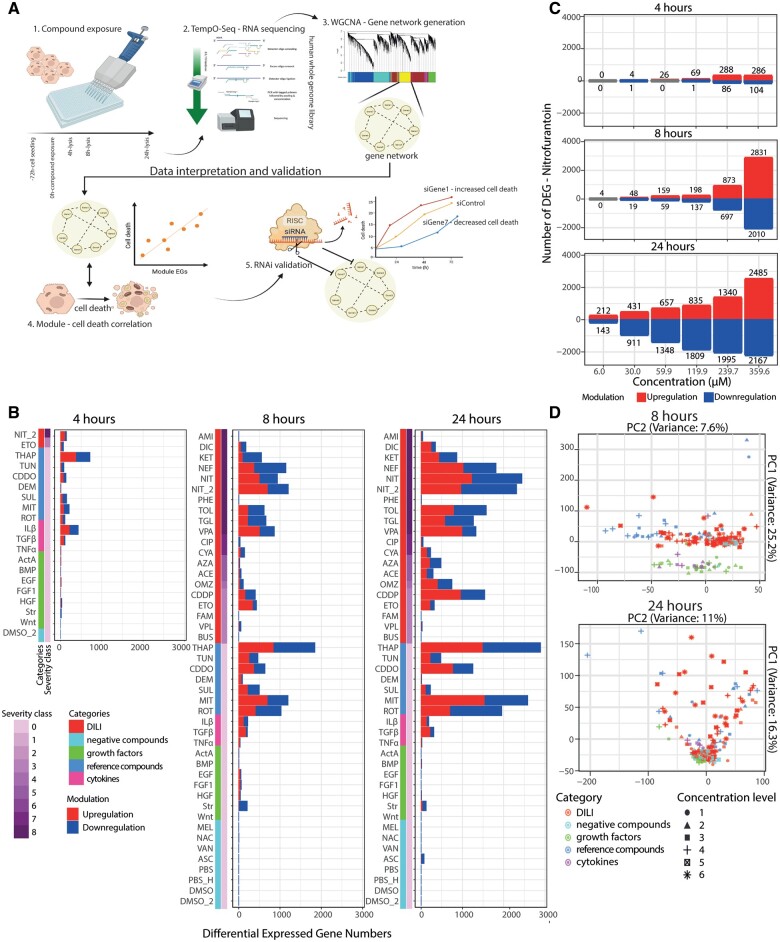
Temporal TempO-Seq whole transcriptome targeted RNA sequencing of HepG2 cells. A, The experimental overview of the study showing the schematic timeline of the cell culture processes to the HTTr method application. The cells were seeded in 384 well plate 72 h before the exposure to the compounds. The cells were lysed and then the lysates were collected for HTTr processes with BioSpyder technology at 4, 8, and 24 h. The lysates were then subjected to the TempO-Seq high throughput RNA sequencing technology deploying the whole genome library. The log2 fold change (FC) values were used to generate gene networks with WGCNA approach. Correlation with the external trait-cell death were performed from the previously generated cell death data (Wijaya *et al.*, 2021). The causal relation of gene memberships from the high cell death-correlated modules with the cell death occurrence were determined using RNAi method. B, The differential expressed gene (DEG) numbers of each tested compound in every time point. Each plot shows the aggregated sum values from all the concentration. The color bars on the rows show compound categories and severity class. In the bars, the blue color indicates the downregulated DEGs and the red color shows the upregulated DEGs. The threshold of the DEGs is set with adjusted *p* value < .01 and log2 FC > [0.1]. C, The differential expression gene numbers of the cells exposed to nitrofurantoin from the lowest dose level (1) until the highest dose level (6) in every time point. The red color of the plot indicates upregulated DEGs and blue color indicates downregulated DEGs. The threshold of the DEGs is set with adjusted *p* value < .01 and log2 FC > [0.1]. D, The PCA plots derived from the log2 FC values, per time point. Every dot of the plots indicates the position of each sample in the plot where the colors indicate the compound category and the shapes represent the dose levels.

## Materials and methods

###  

####  

##### Chemical and reagents

All chemicals were purchased from Sigma-Aldrich—The Netherlands; except for cisplatin (Ebewe—The Netherlands) and nefazodone (Sequoia Research Products—Pangbourne, United Kingdom). All compounds were dissolved in DMSO; except for mitomycin-C (DMSO-PBS) and *N*-acetylcysteine (PBS) and for cisplatin which was already manufactured as a solution. All compounds in DMSO were maintained as 500-fold stock such that the final exposure did not exceed 0.2% v/v DMSO. The cytokines and growth factors were dissolved according to the manufacture protocols. TNFα was purchased from R&D System (Abingdon, United Kingdom), TGFβ was purchased from Immunotools (Friesoythe, Germany), activinA, BMP4, hHGF, hFGFi, hIL-1β, and WNT3a were purchased from PeproTech (London, United Kingdom), human EGF was purchased from Sigma-Aldrich (The Netherlands). PowerUp SYBR green real-time PCR master mix was purchased from ThermoFisher.

##### Cell culture and treatment

Human hepatoma (HepG2) cells were purchased from ATCC—Germany (clone HB8065) and maintained in DMEM high glucose (Fisher Scientific—Bleiswijk, The Netherland) supplemented with 10% (v/v) FBS (Fisher Scientific—Bleiswijk, The Netherlands), 250 U/ml penicillin and 25 µg/ml streptomycin (Fisher Scientific—Bleiswijk, The Netherlands) in humidified atmosphere at 37°C and 5% CO_2_/air mixture. The cells were used between passage 14 and 20. The cells were seeded in Greiner black µ-clear 384 well plates, at 8000 cells per well for the exposure experiment. Unless differently mentioned, we use wild-type HepG2 for the experiments.

The exposure to the compounds and the RNA sequencing was performed at different moments and referred to as batch 1 and batch 2. The first batch was performed for all DILI compounds and the second batch was performed for all cellular stress response reference compounds, cytokines, and growth factors; nitrofurantoin was included in both batches at the same concentrations to assess interbatch variation. The DILI severity of the compounds was adapted from the FDA (Chen *et al.*, 2016). At the day of exposure (3 days after seeding), the compounds were diluted in the culturing medium to meet the final concentration as indicated in Table 1. The plates were incubated in humidified atmosphere at 37°C and 5% CO_2_/air mixture. At the designated time points (4 h—first and second batch, 8 h, and 24 h—second batch), the cells were lysed. The exposure medium was aspirated and the cells were washed with PBS 1 time followed by addition of 25 µl 1× BNN lysis buffer (BioClavis, Glasgow, Scotland) diluted in PBS. The plates were incubated for 15 min at room temperature to enhance the lysis processes. After 15 min incubation, the plates were sealed with an aluminum seal and stored at −80°C and shipped to BioClavis for RNA sequencing. All exposures were performed 3 times independently to cover biological variability. The RNA sequencing experiment was performed 3 times covering 3 biological replicates.

**Table 1.** List of the tested compounds with the supporting information

Compound list (concentration unit)	Abbreviation	Compound category (severity)	Concentration level						Experiment batch
			1	2	3	4	5	6	
Amiodarone (µM)	AMI	DILI compound (8)	0.8	4.0	8.1	16.2	32.3	64.6	1
Diclofenac (µM)	DIC	DILI compound (8)	10.1	50.5	101.0	202.0	404.0	606.0	1
Ketoconazole (µM)	KET	DILI compound (8)	3.3	6.6	33.0	65.9	131.9	263.8	1
Nefazodone (µM)	NEF	DILI compound(8)	1.9	3.9	19.7	39.5	79.0	100.0	1
Nitrofurantoin (µM)	NIT	DILI compound (8)	6.0	30.0	59.9	119.9	239.7	359.6	1
Nitrofurantoin (µM)	NIT2	DILI compound (8)	6.0	30.0	59.9	119.9	239.7	359.6	2
Phenytoin (µM)	PHE	DILI compound (8)	10.9	54.3	108.7	217.3	869.2	1086.5	1
Tolcapone (µM)	TOL	DILI compound (8)	10.9	22.0	109.9	219.9	439.7	879.4	1
Troglitazone (µM)	TGL	DILI compound (8)	6.4	31.8	63.6	127.1	254.2	381.3	1
Valproat (µM)	VPA	DILI compound (8)	121.4	606.9	1213.8	2427.5	4855.1	7282.6	1
Ciproflaxin (µM)	CIP	DILI compound (7)	3.3	16.5	32.9	65.8	131.6	197.4	1
Cyclosporin A (µM)	CYA	DILI compound (7)	0.2	1.0	4.0	8.0	12.0	16.0	1
Azathioprine (µM)	AZA	DILI compound (5)	0.3	1.7	3.4	6.8	20.4	27.2	1
Acetamonophen (µM)	ACE	DILI compound (5)	69.5	347.4	694.7	1389.5	2778.9	4168.4	1
Omeprazole(µM)	OMZ	DILI compound (4)	4.7	23.5	47.0	94.0	188.0	282.1	1
Cisplatin (µM)	CDDP	DILI compound (3)	1.0	2.5	5.0	10.0	15.0	20.0	1
Etoposide (µM)	ETO	DILI compound (3)	1.0	5.0	25.0	50.0			2
Famotidine (µM)	FAM	DILI compound (3)	0.8	4.1	8.2	16.3	32.6	48.9	1
Verapamil (µM)	VPL	DILI compound (3)	0.5	2.6	5.1	10.2	30.5	50.9	1
Buspirone (µM)	BUS	DILI compound (3)	0.0	0.1	0.2	0.3	0.7	1.0	1
Thapsigargine (µM)	THAP	ER stress inducer	0.1	0.5	2.5	5.0			2
Tunicamycin (µM)	TUN	ER stress inducer	0.2	1.0	5.0	10.0			2
CDDO (µM)	CDDO	Oxidative stress inducer	0.0	0.1	0.5	1.0			2
Diethyl maleate (µM)	DEM	Oxidative stress inducer	3.0	16.0	80.0	160.0			2
Sulforaphene (µM)	SUL	Oxidative stress inducer	0.6	3.0	15.0	30.0			2
Mitomycin C (µM)	MIT	DNA damage inducer	1.2	6.0	30.0	60.0			2
Rotenone (µM)	ROT	Mitochondria toxicant	0.0	0.1	0.4	2.0			2
IL-1β (ng/ml)	ILβ	Cytokines	2.0	10.0	50.0	200.0			2
TGFβ1 (ng/ml)	TGFβ	Cytokines	0.5	2.0	10.0	40.0			2
TNFα (ng/ml)	TNFα	Cytokines	0.2	1.0	5.0	20.0			2
Activin A (ng/ml)	ActA	growth factors	0.2	1.0	5.0	20.0			2
BMP4 (ng/ml)	BMP	growth factors	0.2	1.0	5.0	20.0			2
EGF (ng/ml)	EGF	growth factors	0.2	1.0	5.0	20.0			2
FGF1 (ng/ml)	FGF1	growth factors	0.5	2.5	12.5	50.0			2
HGF (ng/ml)	HGF	growth factors	0.5	2.0	10.0	40.0			2
Serum (FBS) starvation (%)	Str	growth factors	0.0	0.1	0.5	2.0			2
Wnt3a (ng/ml)	Wnt	growth factors	0.2	1.0	5.0	20.0			2
Melatonin (µM)	MEL	Negative compound	2.6	12.9	25.8	51.7	103.3	155.0	1
*N*-Acetylcysteine (µM)	NAC	Negative compound	1000.0	2000.0	4000.0	6000.0	8000.0	10000.0	1
Vancomycin (µM)	VAN	Negative compound	1.4	6.9	13.8	27.6	55.2	82.8	1
Vitamin C (µM)	ASC	Negative compound	70.0	349.8	699.5	1399.1			1

The severity of the DILI compounds are defined from a previous study (Chen *et al.*, 2016).

##### RNA sequencing and transcriptomic data analysis

The sequencing was performed deploying the human whole transcriptome library. The probe alignment for whole transcriptome gene set was performed by BioClavis. Briefly, FASTQ files were aligned using Bowtie, allowing for up to 2 mismatching in the target sequence. This pipeline applies several quality controls with mapped/unmapped reads, replicate clustering, and sample clustering (Yeakley *et al.*, 2017). Furthermore, we performed sample quality control steps to exclude samples with lower quality of the sequencing outcome (Supplementary Figure 1). Only the correctly mapped counts (raw count values) were further processed. We first excluded samples with library size (the sum of raw count values) lower than 500 000 counts. Then, the raw count values were normalized with the CPM method. In addition, the replicate correlation for each sample toward the mean of the same conditions was evaluated and the samples showing lower than 0.95 Pearson correlation values were eliminated. The samples passed these QC steps were further used for log2 fold change (FC) calculation (Love *et al.*, 2021). For the log2 FC calculation, the normalized count values of each probe from the treated samples were compared with the values of the DMSO (except for cisplatin, *N*-acetylcysteine, cytokines, growth factors, and solvents [DMSO, DMSO2, PBS, and PBSh]—compared with PBS [12%], PBSh [20%], and DMEM, respectively) from the same time point. The log2 FC values were then used as input for the WGCNA. Complementary, we defined the numbers of the differentially expressed genes using the thresholds: log2 FC > |0.1| and adj-*p* value < .01. The transcriptomic data have been made available on https://www.ebi.ac.uk/fg/annotare/, with accession number E-MTAB-11555 for the first transcriptomic batch and E-MTAB-11578 for the second transcriptomic batch.

##### Gene network generation using the weighted gene coregulated network analysis

Prior to applying the WGCNA approach to the processed data, the most significant probes based on the adjusted *p* values were selected resulting in the 1 probe measurement for each gene. Furthermore, the goodSampleGenes (Langfelder and Horvath, 2008) function was applied to the data matrix to eliminate the gene with nonmeaningful log2 FC. Finally, to identify coexpressed genes from the PHH data, we used the WGCNA R package version 1.51 (Langfelder and Horvath, 2008) and applied it to a matrix consisting of 260 rows (experimental conditions being a combination of compound-time-dose) and 11 153 columns (log2 FC values for genes). We generated unsigned gene modules (enabling the clustering of coinduced and corepressed genes), and selected the optimal soft power threshold maximizing both the scale-free network topology using standard power-law plotting tool in WGCNA. We selected 9 as the optimal soft-power parameter (Supplementary Figure 3A). For each experiment, the EG score (or module score) was calculated which was derived from the log2 FC values of their composing genes (Supplementary Table 1). Briefly, this protocol consisted of performing PCA on the gene matrix of each module, normalizing the log2 FC across the entire dataset using *Z*-score conversion, the first principal component corresponds to the EGs (Sutherland *et al.*, 2018). To facilitate the comparison between modules across treatment, the raw module score was normalized to unit variance (fraction between each module score and its standard deviation across the entire dataset). The EGs indicated the magnitude of activation or repression induced by a given treatment (Supplementary Table 2). We further refined the modules built by merging similar modules (those having correlation of their EGs ≥ 0.8) and obtained 288 modules (Supplementary Figure 2B). Modules were annotated for their cellular responses based on the GO database by performing gene set enrichment using the enrichmentAnalysis function (Langfelder and Horvath, 2008) (Supplementary Table 3). The most significant annotation based on the adjusted *p* values was then chosen to represent the cellular responses of the modules. For every gene in a module, the correlation was calculated between the log2 FC versus the EG score of its parent module across the 260 experiments (termed “corEG”). The gene with the highest correlation (so-called “hub gene”) was the most representative of the entire module matrix and show stronger connection to the other gene memberships (Supplementary Table 1). Preservation between the modules structure obtained with the HepG2 data set and the PHH TG-GATEs was performed using preservation statistics calculated with the WGCNA R package and thresholds for interpretation were adopted from the relevant literature (Langfelder *et al.*, 2011). Module showing *Z* summary >= 2 was considered moderately preserved, >= 10 highly preserved. A lower median rank indicates higher preservation.

##### Correlation analysis between module activation and cell death outcomes

The correlation analysis was applied to the module scores and the cell death outcomes from the previous study (Wijaya *et al.*, 2021). The cell death data were derived using live cell imaging capturing the fraction of the cell death indicated by with the propidium iodide (PI) staining for necrosis and annexin V (AnV) staining for apoptosis. The cell death datasets at 8, 24, 48, and 58 h were selected for the correlation analysis. The data points with shared exposure conditions (the same compound and concentration) in both module scores and cell death outcome were selected. The correlation analysis was applied to each compound for every cell death type (necrosis and apoptosis) from the previously generated cell death data (Wijaya *et al.*, 2021) using Pearson correlation method in which the correlation value was composed of the outcomes from the shared concentration from both dataset. Using these shared datapoints, the correlation analysis was performed in every time point of cell death measurement for every compounds expressed as module responses (for all time points of transcriptomics measurement) in *x*-axis and cell death responses at a particular time point (8, 24, 48, or 58 h) in *y*-axis. The positive correlated modules were then selected with the thresholds of correlation adjusted *p* values < .1, correlation score > 0.5, EGs > 2 at least in 1 data point, and > 4 DILI compounds in which the correlation outcomes passing the thresholds. The genes inside the modules for further validation were selected based on the thresholds of adjusted *p* values < .1 and log2 FC > 2 at least in 1 condition.

##### RNA interference screen and live cell imaging for cell death determination

Among the high upregulated gene memberships of the high cell death-correlated modules, we selected the 67 target genes based on the availability of the siRNAs from the drugable genome library. siRNAs against targeted genes were purchased from Dharmacon (ThermoFisher Scientific) as siGENOME SMARTpool reagents, as well as in the form of individual siRNAs (Supplementary Table 4). HepG2 cell suspensions were transiently transfected with the mixture of siRNAs (50 nM) and INTERFERin (Polyplus) in DMEM high glucose and seeded in Greiner black µ-clear 96-well plates, at 25 000 cells per well. The medium was refreshed 24 h post-transfection and compound exposures were performed 48 h afterward. siGENOME nontargeting pool 1 (siNo1) and mock (INTERFERin containing medium) condition were used as the control. On the day of the exposure, the cells were incubated with medium containing 100 ng/ml live Hoechst for 2 h. The medium was then refreshed with the medium containing 0.2 µM PI and 1:2000 AnV-Alexa 633. Sequentially, the compounds were added to the plate to reach the desired concentration. For the RNAi experiment, we used nitrofurantoin (360–480 µM) and nefazodone (39.5–79 µM). The plates were imaged at 24, 48, and 72 h after exposures using a Nikon TiE2000 confocal laser microscope (laser: 647, 540, and 408 nm), equipped with automated stage and perfect focus system. During the imaging, the plates were maintained in humidified atmosphere at 37°C and 5% CO_2_/air mixture. The imaging was performed with 20× magnification objective. The siRNA experiment was performed 3 times covering 3 different biological replicates. To ensure the efficiency of the overall knockdown, we incorporated previously established HepG2-CHOP-GFP (Wink *et al.*, 2017) in the RNAi experiment. We perturbed the expression of CHOP-GFP using siDDIT3 (and siNo1 as a control) following by exposing these cells to tunicamycin (60 µM). The expression of the CHOP-GFP was followed for 48 h using previously mentioned imaging procedures.

##### Image analysis

The images were manually sorted to exclude images which did not fulfil the criteria for analysis: nonbiological background (unanticipated auto fluorescent particulate matter), loss of nuclear signal, and out-of-focus images. The quantitative image analysis was performed with ImageJ version 1.52p and CellProfiler version 2.2.0. First, the nuclei per image were segmented with watershed masked algorithm on ImageJ and thereafter processed with an in house developed CellProfiler module (Wink *et al.*, 2017; Yan and Verbeek, 2012). The results were stored as HDF5 files. Data analysis, quality control, and graphics were performed using the in-house developed R package h5CellProfiler. The nuclear Hoechst33342 intensity levels, nuclear area, PI area, and AnV area were measured at the single cell level. The number of PI and AnV-positive cells were determined based on the count of cells with higher than 10% overlapping between nuclear area and PI/AnV area. For the RNAi experiments, the *z*-score of the cell death was calculated with this formula:

$$Z=\frac{x-\mu }{\sigma },$$

where x is the cell death value of each siRNA; μ is the mean of the population (from the same compound, time point, and cell death type); σ is the standard deviation of the population.

##### Data representation

The graphical representation of the results were generated or modified with Illustrator CS6 and R (ggplot2 [Wickham *et al.*, 2016] and pheatmap [Kolder R, 2019]). Correlation analysis was performed with the R function from the package Hmisc (Harrel, 2021). The hierarchical clustering is performed with “*Ward D2*” algorithm applied to the Euclidian distance between measured variables. Specifically for compound clustering based on the module activity, the clustering was subjected to cutree() function at the height of 20 to define the groups.

## Results

###  

#### Transcriptomic perturbations in the HepG2 cell line exhibit different response patterns between DILI and non-DILI compounds

We performed an extensive toxicogenomic study in HepG2 cells exposed to different DILI compounds at different concentrations for varying time points (Table 1). We also included several cytokines and growth factors as well as defined reference compounds for toxicity relevant cellular stress response pathways to both broaden the diversity of xenobiotic-induced transcriptional responses and to provide benchmark information on important cellular responses to stress. Several compounds were also added as negative controls that showed no cellular stress response modulation in HepG2 cells in previous studies and have no DILI liability (Wijaya *et al.*, 2021). Samples were processed for high-throughput transcriptomic (HTTr) based on targeted whole genome RNA sequencing using the TempO-Seq technology. TempO-Seq data showed overall sufficient library size across as well as replicate correlation for the different treatments (Supplementary Figure 1). Differential expressed genes (DEGs) for each condition were determined with a defined threshold: adjusted *p* values < .01 and log2 FC > |0.1| cutoff. The aggregated number of DEGs, based on the sum of the unique DEGs for all concentrations from each compound, reflected stronger transcriptional responses by the various DILI and reference compounds, but more limited responses for cytokines and growth factors (Figure 1B). Comparing responses between 2 separate experiments (batches) with nitrofurantoin (NIT and NIT2) indicated that the interbatch variation with respect to the number of DEGs and fold change was minimal (Figure 1B and Supplementary Figure 2A). Furthermore, most transcriptomic responses induced by the DILI compounds and positive reference compounds showed clear time dependence (Figure 1B) with strongest response observed at 24 h. However, the transcriptomic responses of the cytokines and growth factors were more prominent at 8 h (Supplementary Figure 2B—examples FGF1, HGF, and EGF). In addition, the transcriptomic perturbations exhibited a concentration dependence with higher numbers of DEGs at increasing concentration of DILI compounds (Figure 1C—exemplified for nitrofurantoin). Additionally, the principal component analysis plots showed different clusters of samples based on the compound categories, time points, and concentration level, indicating different transcriptomic profiles upon the exposure of the different compound categories at different concentration and time points (Figure 1D). Consistent with the number of DEGs, most of the DILI compounds and positive compounds at the higher dose (dose level >= 4) exhibited clear separation from negative compounds (24 h PCA). The responses of the cytokines and growth factors clustered separately from other compounds at 8 h but not at 24 h, confirming the early transcriptomic perturbation. These findings suggest that each compound category did induce distinctive transcriptomic responses. Overall, the thus established gene-level data suggested that the compound sets used induced robust gene expression responses with considerable diversity amongst treatments.

#### Cellular stress response-related gene networks show coherent modulation upon the exposure of positive control compounds

Previous work has shown that coexpression analysis can yield both expected as well as novel insights into biological responses to cellular stress (Callegaro *et al.*, 2021, 2023; Sutherland *et al.*, 2018). In order to identify gene networks associated with cellular responses, we performed WGCNA on the transcriptomic data to identify coregulated sets of genes (modules). We obtained 288 modules compose of 14 359 genes (Supplementary Figure 3B and Supplementary Table 1) which resulted in 98% reduction of the dimensionality of the gene expression originally derived from the readout of approximately 21 111 probes. Moreover, the WGCNA also reduced the interbatch variation as indicated by the higher correlation of the transcriptional changes upon nitrofurantoin treatment at the module level (Pearson *R*: 0.94) compared with the gene level (Pearson *R*: 0.74). The batch correlation at the module level was comparable with the correlation at the DEGs level (Pearson *R*: 0.96) (Supplementary Figure 2A). This indicated that the module network derived from WGCNA was able to reduce dimensionality and eliminate the noise effect due to non-perturbed and/or low expressed genes.

**Figure 2.**
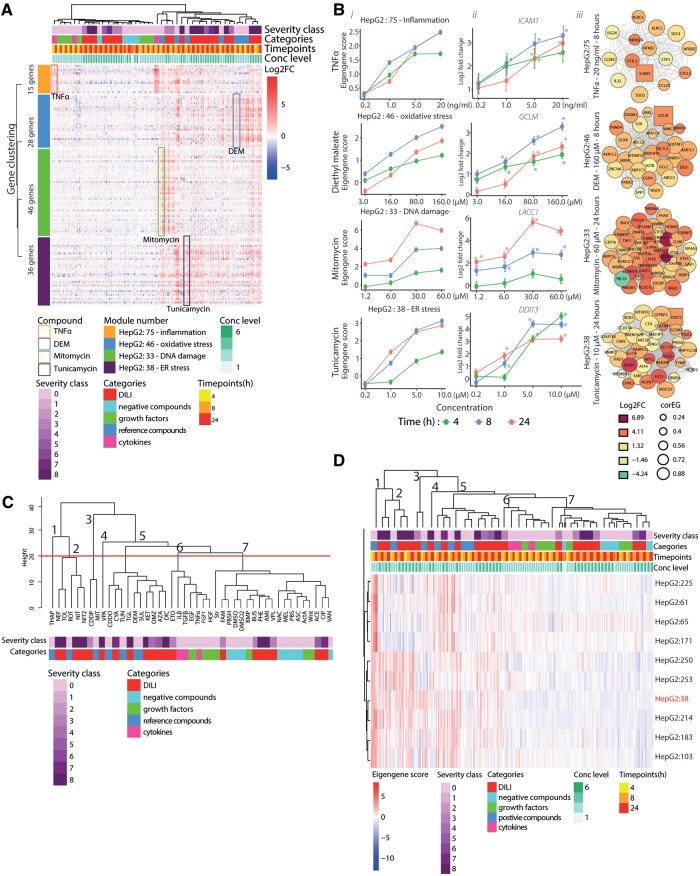
Gene network activation of WGCNA-based modules by DILI compounds. A, A heatmap (*n* = 3) exhibiting the modulation of the gene memberships of HepG2:75, HepG2:46, HepG2:33, and HepG2:38. The highlighted parts (colored boxes) show the modulation of the gene upon the positive control exposure: orange—TNFα, blue—DEM, green—mitomycin, purple—tunicamycin. The heatmap contains 4 variables on the columns indicated by distinctive color groups: severity class of the compound, compound categories, time points, and dose level. These variables describe the exposure condition applied to the cells. Each row of the heatmap shows the log2 FC values of each module memberships from every sample where red color shows upregulation and blue color shows downregulation. The color on the row indicates the modules (orange: HepG2:75, blue: HepG2:46, green: WGCNA: HepG2:33, and purple: HepG2:38). The clustering of the heatmap is performed using the “*Ward d2*” algorithm applied to the Euclidian distance between aggregated variables (column clustering: mean of the log2 FC values of the dose levels and time points per compound, row clustering: mean of the log2 FC values of the genes per module). B, The dose response plots of stress response-related modules upon the exposure of the specific reference compounds: HepG2:75—inflammation (TNFα), HepG2:46—oxidative stress (DEM), HepG2:33—DNA damage (etoposide), HepG2:38—ER stress (tunicamycin) (i). The dose-response plots of the hub gene of each stress response related module upon the exposure of the specific reference compounds: HepG2:75—*ICAM1*, HepG2:46—*GCLM*, HepG2:33—*TNFRS10A*, HepG2:38—*MTFD2*. The color of the plot represent the time points, the error bars indicate standard error mean (SEM) values, *n* = 3. Stars indicate the significant upregulation with adjusted *p* value < .01 (ii). The overview of the gene memberships’ activities of the stress response modules upon the exposure of the positive compound at the particular time point and concentration. The box node indicate the hub gene (the gene with the highest correlation to the parent module). The color of the nodes represents the modulation and the size of the nodes represent the module correlation (iii). C, The compound clustering based on aggregated mean eigengene (EG) scores for every time point and dose level per compound. The hierarchical clustering is performed using “*Ward D2*” algorithm applied to the Euclidian distant between aggregated values. The compounds are annotated with the severity and categories showed by the color bars. The determination of the clusters was set at the height 20 with the cuttree() function. D, A heatmap showing the cluster of the modules annotated as the endoplasmic reticulum activities and ER stress responses. The heatmap contains 4 variables indicated by distinctive color groups: severity class of the compound, compound categories, time points, and dose level. These variables describe the exposure condition applied to the cells. Each row of the heatmap shows the EG values where red color shows activated and blue color shows deactivation. The clustering of the columns is performed with the same manner as compound clustering (C). HepG2:38-ATF-CHOP ER stress is highlighted with red.

Because our goal was to identify causal links among genes, biological responses and liver injury using HepG2 as an *in vitro* model, we first aimed to validate the approach by determining whether expected stress responses for selected reference compounds were reflected in gene networks and their biological annotations. We focused on the activity of the modules that were annotated for biological responses know to be associated with DILI-related cellular stress responses (Weaver *et al.*, 2020; Wink *et al.*, 2018): Module annotations were based on the gene ontology enrichment results for each individual module (Supplementary Table 3) and supported by the presence of well-established gene memberships in the adaptive stress responses. Using this approach, we selected 4 modules for further analysis: HepG2:75-inflammation, HepG2:46-oxidative stress, HepG2:33-DNA damage, and HepG2:38-ATF4-CHOP complex (ER stress related). The latter module is a generalized stress response target by the EIF2-alpha kinases often associated via CHOP/DDIT3 as a downstream effector arm of ER stress (Yang *et al.*, 2020). As expected, genes belonging to the same modules showed similar regulation patterns indicative of coregulation while responses between genes from different modules varied (Figure 2A). This effect was particularly appreciable upon the exposure to the reference compound: TNFα for HepG2:75-inflammation, DEM for HepG2:46-oxidative stress, mitomycin C for HepG2:33-DNA damage, and tunicamycin for HepG2:38-ER stress (Figure 2A). Thus, individual HepG2 module genes did respond in a coordinated fashion to compounds expected to activate the biological processes derived empirically solely from the module enrichment annotations.

Next, we asked whether the module EGs score, which is a single value reflecting the behavior or all module genes, responded in a similar manner using these 4 reference compounds. We compared the EGS to the fold change for the module hub gene (the gene with the highest module correlation or Core EGS; Supplementary Table 1). The stress response module EGS showed time- and concentration-dependent responses; module activity was typically already high at 8 h, except for HepG2:33 which showed strongest induction at 24 h. As expected, the upregulation of the hub genes for each of these 4 modules showed a similar concentration and temporal response pattern to their parent modules (Figure 2Bii). The modulation of the gene members for each stress response related module was illustrated in the arrangement of nodular networks with the square node as the hub gene (Figure 2Biii). Yet, *DDIT3*, the HepG2:38 hub gene, showed strong upregulation already at 4 h with a peak at 8 h. However, we observed that >50% of the HepG2:38 gene members showed stronger upregulation at 24 h, significantly contributing to the linear time dependency of the parent module (Supplementary Figure 4A). Based on these findings, we conclude that the modulation of the different cellular stress responses, based on the module enrichment, is reflected by the module EGS and the member genes exhibited the expected coherent activation patterns after treatment.

#### Coexpression modules capture compound class differences and highlight candidate cellular mechanisms underlying high DILI risk compounds

We further interrogated the activity of the modules beyond the stress response-related modules in order to uncover mechanisms in HepG2 cells activated after DILI compound exposure. Hierarchical cluster analysis revealed that both compounds and modules clustered according to the EGS activity (Supplementary Figure 4B). There were 7 compound clusters (Figure 2C): clusters 1–5 contained the reference compounds and DILI compounds with high DILI severity class, cluster 6 consisted of the cytokines and growth factors, clusters 7 mainly included the low severity class DILI compounds as well as DILI negative compounds. Overall, compound clusters 1–5 showed stronger (de)activation of the various modules compared with clusters 6–8 (Supplementary Figure 4B). Of note, acetaminophen was grouped in cluster 8 and showed minor module activation, which was anticipated because HepG2 cells have little cytochromeP450 2E1 (Hiemstra *et al.*, 2019) required for the formation of the toxic metabolite. As an example, for the module clustering, we highlighted 1 module cluster (see red box in Supplementary Figure 4B and detailed in Figure 2D). Interestingly, this cluster did consist of the modules annotated for the endoplasmic reticulum-related responses or/and stress (including HepG2:38—red, HepG2:61, HepG2:225, HepG2:65, HepG2:171); modules in this clusters were strongly activated by high severity DILI compounds (clusters 1–4) including nefazodone, tolcapone, and nitrofurantoin (Figure 2D). We also identified module clusters of DNA damage responses and mitochondria membrane potential to be strongly modulated by high severity DILI compounds.

We then identified the most activated and repressed modules (Table 2) across the entire compound set to investigate the strongest modulated cellular mechanisms upon DILI compound treatment. In order to select strongly perturbed modules, the median scores of the EGS across the conditions were calculated. We further counted the number of conditions that perturbed the modules resulting in EGS > 2 for activation and EGS < −2 for repression. Based on these 2 criteria, the modules were sorted, and the top 20 activated and repressed modules were selected. Interestingly, all stress response-related modules were among the top 20 activated modules along with other ER stress related (HepG2:38) and inflammation related responses (HepG2:140). Moreover, cytoskeletal reorganization (HepG2:101, HepG2:158, HepG2:196) and small molecule activity (HepG2:206, HepG2:235) were also highly activated. On the other hand, the top 20 top repressed modules consisted of the modules annotated for organelle biogenesis (HepG2:24, HepG2:37, HepG2:71), mitochondria (HepG2:70, HepG2:282), metabolism activity (HepG2:58, HepG2:251), and cell cycle activation (HepG2:10, HepG2:252). Altogether, these coexpression modules provide mechanistic insight in the cellular processes that are involved in the hepatocellular mechanisms of DILI.

**Table 2.** Top 20 most modulated gene networks upon the exposures of the tested compounds

Module	Modulation	Annotation	Hub gene	Preservation in PHH
HepG2:1	Deactivated	G-protein-coupled receptor activity, transmembrane signaling receptor activity, signaling receptor activity	*KRTAP16-1*	Nonpreserved
HepG2:4	Deactivated	Polyketide metabolic process	*GJB1*	Preserved
HepG2:10	Deactivated	Cell cycle process, DNA replication, DNA metabolic process, mitotic cell cycle process	*SAC3D1*	Preserved
HepG2:24	Deactivated	Nucleosome assembly, chromatin assembly	*HIST1H2AD*	Preserved
HepG2:32	Deactivated	Positive regulation of glycogen biosynthetic process, leukocyte activation involved in inflammatory response	*ZNF607*	Preserved
HepG2:36	Deactivated	Rough endoplasmic reticulum lumen, DNA binding	*ZNF594*	Preserved
HepG2:37	Deactivated	Positive regulation of mitochondrial membrane potential	*MSS51*	Nonpreserved
HepG2:58	Deactivated	Cholesterol biosynthetic process	*MVD*	Preserved
HepG2:69	Deactivated	Thyroid-stimulating hormone	*FOXD4L3*	Nonpreserved
HepG2:70	Deactivated	Mitochondrial membrane part, integral component of organelle membrane	*COA3*	Preserved
HepG2:71	Deactivated	Endoplasmic reticulum lumen, posttranslational protein modification, histamine metabolic process	*SERPINA10*	Preserved
HepG2:86	Deactivated	Protein localization to plasma membrane, COPII adaptor activity	*TEX261*	Nonpreserved
HepG2:123	Deactivated	Procollagen-proline 4-dioxygenase activity, oxidoreductase activity	*EFCAB3*	Nonpreserved
HepG2:137	Deactivated	Positive regulation of cellular response to hypoxia, hypoxia-inducible factor-1alpha signaling pathway	*RNF170*	Nonpreserved
HepG2:147	Deactivated	Endoplasmic reticulum-Golgi intermediate compartment, endoplasmic reticulum membrane, MHC class I protein complex assembly	*HINT2*	Preserved
HepG2:150	Deactivated	Xylulokinase activity, IkappaB kinase complex binding	*LARS2*	Nonpreserved
HepG2:175	Deactivated	RNA methyltransferase activity, cellular response to peptide, DNA-dependent ATPase activity	*METTL3*	Nonpreserved
HepG2:251	Deactivated	UDP catabolic process, pyrimidine nucleoside diphosphate catabolic process	*JRK*	Nonpreserved
HepG2:252	Deactivated	Positive regulation of cell cycle, nuclear division, organelle fision, cell division	*CCNF*	Preserved
HepG2:282	Deactivated	Mitochondrial membrane fusion, desmosome assembly, positive regulation of mitochondrial translation	*RCC1L*	Nonpreserved
HepG2:3	Activated	Regulation of signaling, regulation of cell communication, regulation of response to stimulus, regulation of signal transduction	*UBQLN1*	Preserved
HepG2:33	Activated	Signal transduction by p53 class mediator, apoptotic process, cellular response to UV	*TNFRSF10A*	Preserved
HepG2:38	Activated	CHOP-ATF4 activity, ER stress response, ER stress-related apoptotsis	*MTHFD2*	Preserved
HepG2:43	Activated	Negative regulation of blood vessel remodeling	*PALM2*	Nonpreserved
HepG2:46	Activated	Oxidative stress, polyketide metabolic process, alditol: NADP+ 1-oxidoreductase activity	*GCLM*	Preserved
HepG2:52	Activated	DNA-binding transcription repressor activity, RNA polymerase II specific, regulation of cellular metabolic process	*IBTK*	Preserved
HepG2:61	Activated	Response to endoplasmic reticulum stress, endoplasmic reticulum unfolded protein response, IRE1-mediated unfolded protein response	*DNAJB9*	Preserved
HepG2:75	Activated	Response to cytokine	*ICAM1*	Preserved
HepG2:83	Activated	Synapse assembly, positive regulation of transcription, DNA templated	*PIEZO1*	Preserved
HepG2:84	Activated	Tubulin-tyrosine ligase activity, C-terminal protein-tyrosinylation, histidine-tRNA ligase activity	*SLC25A51*	Nonpreserved
HepG2:101	Activated	Piccolo NuA4 histone acetyltransferase complex, nuclear part, prenyltransferase activity	*FOXQ1*	Nonpreserved
HepG2:112	Activated	KDEL sequence binding, autophagosome, TGFb receptor signaling activity	*PIP4K2C*	Preserved
HepG2:140	Activated	Notochord cell differentiation	*NDE1*	Nonpreserved
HepG2:158	Activated	Cadherin binding, cell adhesion molecule binding	*SNHG8*	Preserved
HepG2:196	Activated	Actin cytoskeleton, homotypic cell-cell adhesion	*TAGLN*	Preserved
HepG2:206	Activated	Multicellular organism aging, aspartate-glutamate transport	*CREBRF*	Preserved
HepG2:214	Activated	Atg1/ULK1 complex activation, ER stress, macroautophagy	*SESN2*	Preserved
HepG2:228	Activated	Mitochondrion localization, neurotrophin production, negative regulation of PPAR signaling pathways	*REM2*	Nonpreserved
HepG2:235	Activated	Adenylate-cyclase inhibiting adrenergic receptor activity, glycine transport	*CALCOCO2*	Nonpreserved
HepG2:253	Activated	DODECENOYL-COA DELTA-ISOMERASE ACTIVITY	*ERRFI1*	Preserved

The top 20 most repressed and activated upon exposure of the tested compounds. The selection was based on the calculation of the median scores of the EGS across all conditions and the number of conditions (count) which perturb the modules resulting in EGS > 2 for activation and EGS < −2 for repression. Based on these 2 criteria, the modules were sorted and top 20 activated and repressed modules were selected.

#### Stress response-related modules are preserved in primary human hepatocytes

**Figure 3.**
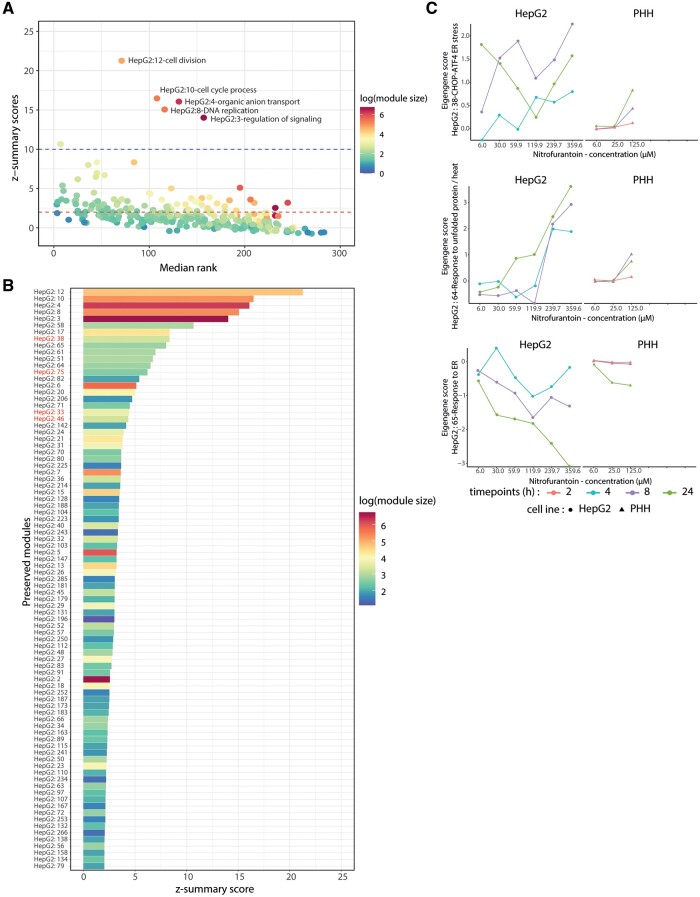
HepG2 module preservation analysis in the primary human hepatocytes (PHH). A, The correlation plot between 2 module preservation parameters: *z-*summary scores and median rank. The red line in the *y*-axis intercept corresponds to *z*-summary score = 2 and the blue line to *z*-summary score = 10. Each dot represents each module in the plot. The color of the plots represents the module size in log scale. B, The list of the HepG2 modules that are preserved in the PHH systems. The module written in red are the stress responses related modules. The color of the bar plots represents the module size in log scale. C, Response of HepG2 and PHH upon nitrofurantoin exposure based on the HepG2:38, HepG2:64, and HepG2:65. The colors of the plot represent time points and the shapes of the dots indicate the cell lines.

Having established that modules reduce the complexity of gene expression data to interpretable mechanistically relevant biological responses, we aimed to determine whether HepG2 modules were preserved in PHH, a gold standard for human liver *in vitro* test systems. We performed a preservation analysis of the HepG2 modules using the TG-GATEs-based PHH modules as a reference (Callegaro *et al.*, 2021) (Figure 3). Based on the *z*-summary preservation scores, 87 modules (approximately 30%) of the HepG2 modules showed moderate (*z*-summary 2–10) to high preservation (*z*-summary >10) in the PHH dataset (Figure 3B). The top 10 most preserved HepG2 modules in PHH were annotated with multiple essential cellular processes and functions such as cell cycle and division (HepG2:12, HepG2:10, HepG2:8), signal transduction (HepG2:3), cellular biosynthesis and respiration (HepG2:58, HepG2:4, HepG2:17), and endoplasmic reticulum-related processes (HepG2:38, HepG2:65, HepG2:61). In addition to these modules, the modules related to cellular stress responses (Hiemstra *et al.*, 2019; Wink *et al.*, 2014, 2017) such as oxidative stress (HepG2:46), DNA damage (HepG2:33), inflammation (HepG2:75), and ATF-4-CHOP complex (HepG2:38) were also preserved in PHH (marked in red). This suggested that the expression of the genes of the aforementioned stress responses modules are similarly coregulated in HepG2 and PHH. The overall module responses of the preserved modules exhibit a similar concentration-response and temporal pattern of (de)activation between HepG2 and PHH (Supplementary Figure 5D).

To examine the similarity of the dynamics of preserved responses across the 2 test systems, we interrogated the dynamics of ER stress-related responses as an example, exemplified with nitrofurantoin treatment (Figure 3C). In order to calculate the different module EGS for PHH, we used as input the log2 FC derived from the responses of PHH as previously reported (Igarashi *et al.*, 2015). Module activation was observed for HepG2:38 and HepG2:64 (responses to unfolded protein and heat); module repression was observed for of HepG2:65 (response to ER stress). The temporal responses across test systems showed strong resemblance (Figure 3C). Yet, modules in HepG2 showed higher absolute EGS values compared with PHH; this was likely due to the higher log2 FC values in HepG2 compared with PHH in relation to the higher compound concentration and the broader dynamic range of RNA sequencing compared with microarray (Zhao *et al.*, 2014) (Supplementary Figure 5A). Overall, for nitrofurantoin exposure, the module responses between HepG2 and PHH exhibited moderate similarity with 0.56 Pearson correlation score when the cells were exposed with comparable conditions (Supplementary Figure 5B). As ER stress showed strong preservation, we evaluated this response based on the 3 preserved modules on another ER stress inducing DILI compound, cyclosporine A. We observed similar direction of module activation between HepG2 and PHH (Supplementary Figure 5C). Interestingly, for cyclosporine A, we found in both cells the activation of HepG:65 while HepG2:64 was not induced. In conclusion, we identified the gene networks that are preserved between HepG2 and PHH, and delineated the relevance of HepG2 observations for PHH.

#### Modules highly correlated to cell death reveal candidate hepatocellular responses networks

**Figure 4.**
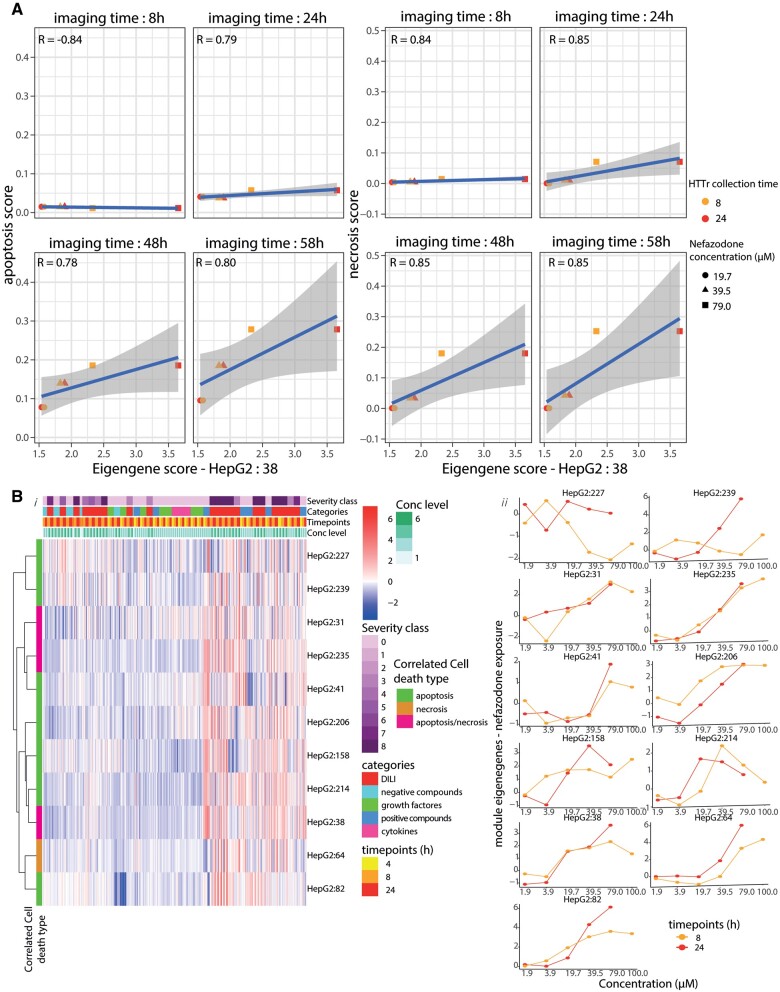
Module correlation analysis with the cell death. A, The correlation plots between EG score of HepG2:38 and percentage apoptosis (left) and necrosis (right) at designated cell death measurement time points (8, 24, 48, and 58 h) on the cells exposed to nefazodone. The color of the dots corresponds to the transcriptomic time point and the shape of the dots to the concentration. B, A heatmap showing the EG scores of high correlated module to the cell death upon the exposure of the compounds. The heatmap contains 4 variables indicated by distinctive color groups: severity class of the compound, compound categories, time points, and dose level. These variables describe the exposure condition applied to the cells. Each row of the heatmap shows the EG values of each module from every sample where each column of the heatmap indicates the sample bearing the identities based on the assigned variables. The modules are clustered using “*Ward D2*” algorithm. The color identity of the module indicates the correlated cell death type. The color of the heatmap representing the modulation of the gene networks (module) where red color shows activated and blue color shows deactivation (i). Dose response plots of the high cell death-correlated modules upon the exposure of nefazodone at 8 (orange) and 24 (red) hours (ii).

As the final step, we aimed to establish direct causal relationships between critical gene module responses and cell death. First, we identified modules that significantly correlated with cell death to assemble a list of candidate modules and associated genes. We identified modules that were linked to hepatocellular death by correlating the EGS of all modules with the live cell imaging of necrotic and apoptotic cell death readouts, propidium iodide, and AnnexinV-Alexa633, respectively (Wijaya *et al.*, 2021). We performed the correlation analysis between modules’ EGS and the cell death outcomes measured at 8, 24, 48, and 58 h, across all the compounds tested in order to identify common responses activated in the HepG2 in relation to cell death. The correlation between early module activities and cell death at 58 h resulted in the highest score of significant correlating pairs (adjusted *p* values < .1; Supplementary Figure 6A). As an example, nefazodone treatment caused most extensive cell death as well as strong transcriptomic perturbations; we observed high correlation between EGS of HepG2:38 and cell death markers at later time points (Figure 4A).

Because cell death onset was observed at late time points, we further focused on the correlation analysis between the module activities (at 4, 8, and 24 h) and cell death measured at 58 h. We identified module correlation scores with cell death outcomes for several DILI compounds (Supplementary Table 5). Eleven modules passed the criteria of correlation adjusted *p* values < .1, correlation score > 0.5, EGs > 2 at least in one data point, and >4 DILI compounds in which the correlation outcomes passed these thresholds (Table 3). Ten of the high cell death modules were correlated with apoptotic and necrotic cell death and interestingly only 1, HepG2:64, was significantly correlated with necrotic cell death. The activities of these modules showed prominent modulation mostly upon the exposure with severe DILI compounds (Figure 4Bi). We observed clear concentration response activation of these modules for the severe DILI compounds (see as example, Figure 4Bii—nefazodone and Supplementary Figure 6B—nitrofurrantoin, troglitazone, and diclofenac). Because most modules associated with cell death were already activated at very early time points, this suggested that the activation of these modules may also be causally associated with later cell death onset in HepG2 cells. The annotation of these modules showed broader cellular responses, some with known involvement in liver injury: ER stress-related responses (HepG2:38, HepG2:64) (Liu and Green, 2019) and cytoskeletal reorganization (HepG2:39, HepG2:158) (Shepard and Tuma, 2010). In addition, cellular processes related to adenylate-cyclase (HepG2:235) were also highly correlated with cell death. Altogether, we identified key modules that exhibited high correlation with the occurrence of cell death.

**Table 3.** Modules with high correlation with cell death

Module	Hub gene	Correlated cell death type	Annotation	Preservation in PHH
HepG2:227	ZDHHC13	Apoptosis	Exocytosis	Nonpreserved
HepG2:239	C18orf21	Apoptosis	DNA endonuclease, demethylation	Nonpreserved
HepG2:31	YWHAB	Apoptosis/necrosis	Nuclear part	Preserved
HepG2:235	CALCOCO2	Apoptosis/necrosis	Adenylate-cyclase inhibiting adrenergic receptor activity	Nonpreserved
HepG2:41	ZBED9	Apoptosis	DNA-binding transcription factor activity	Nonpreserved
HepG2:206	CREBRF	Apoptosis	Multicellular organism aging	Preserved
HepG2:158	SNHG8	Apoptosis	Cadherin binding, cell adhesion molecule binding	Preserved
HepG2:214	SESN2	Apoptosis	Atg1/ULK1 kinase complex, response to endoplasmic reticulum stress	Preserved
HepG2:38	DDIT3	Apoptosis/necrosis	CHOP-ATF4 complex, intrinsic apoptotic signaling pathway in response endoplasmic reticulum stress	Preserved
HepG2 64	HSPH1	Necrosis	Response to unfolded protein, response to heat	Preserved
HepG2:82	MT1M	Apoptosis	Stress response to metal ion	Preserved

These modules pass the threshold of correlation adjusted *p* values < .1, correlation score > 0.5, EGs > 2 at lease in 1 data point, and > 4 DILI compounds in which the correlation outcomes passing the thresholds. The order is based on the clustering on Figure 4Bi.

#### A decrease in cell death is found upon the perturbation of the gene memberships of the high cell death-correlated modules

**Figure 5.**
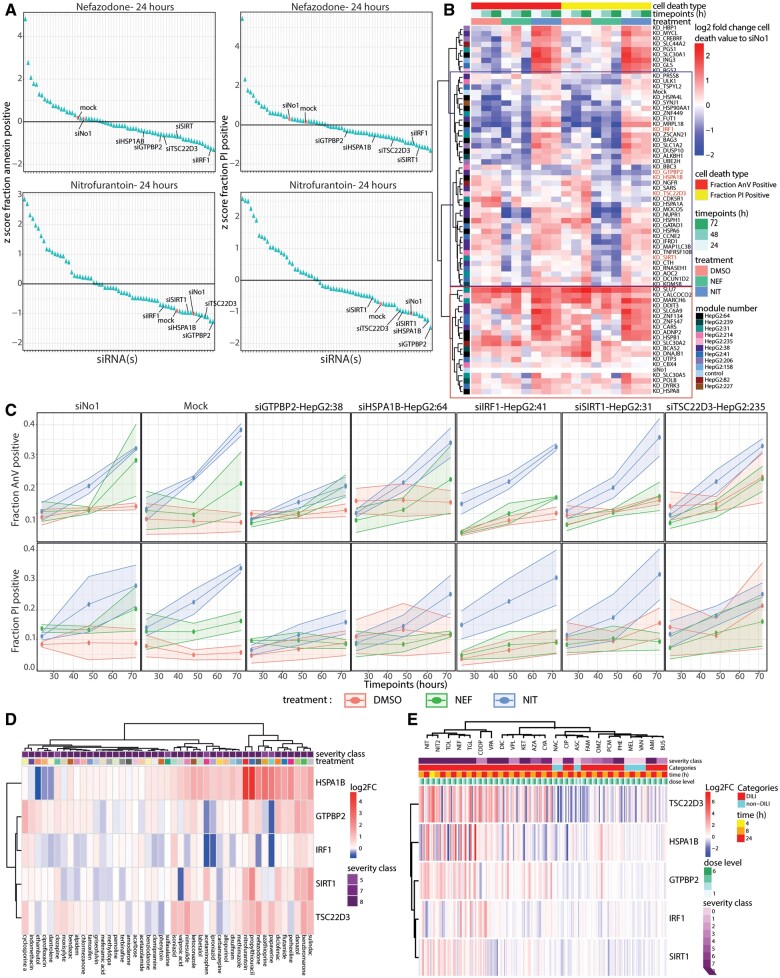
RNA interference to infer causal relationship of module gene memberships with cell death. A, The dot plots showing the *z*-score of the cell death modulation in each perturbation compared upon nefazodone (top) and nitrofurantoin (bottom) exposure at 24 h (left—annexin-V-Alexa633 readout, bottom—PI readout). The colors of the dots represent the type of the perturbation (red: control (mock [medium + INTERFERin] and siNo1 [medium + INTERFERin + scrambled siRNA]), blue: siRNA targeting genes). B, A heatmap (*n* = 3) showing the log2 FC cell death values compared with siNo1 of the HepG2 cells upon siRNA perturbation reducing the expression of the (highly upregulated) genes memberships of the high cell death-correlated modules. The heatmap consists of 3 legends: cell death type (red: fraction annexin-V positive cells; yellow: fraction PI positive cells), imaging time (green intensity: 24, 48, and 72 h), and treatment (orange: DMSO; green: nefazodone [39.5 µM]; blue: nitrofurantoin [360 µM]). The color on the row indicates the parent modules of the perturbed genes. The red intensity of the heatmap positively represents the cell death magnitude. The blue box contains the siRNAs that reduce the cell death and the red box contains the siRNA that increase the cell death. The red siRNAs highlight the highest cell death reduction upon the perturbation of the target genes. C, The detailed overview of the perturbation of the hit genes (in red) resulting in the highest reduction of the cell death (top: fraction annexin-V positive cells, bottom: fraction PI positive cells). The color of the plot indicates the treatment (red: DMSO, green: nefazodone, blue: nitrofurantoin). The shadow of the plot representing the standard error mean (SEM), *n* = 3. D, The heatmap shows the expression in PHH of the 5 genes whose perturbation resulted in the highest reduction of the cell death fraction in HepG2 cells upon the exposure of nitrofurantoin (360 µM) and nefazodone (39.5 µM). The heatmap contains 3 variables on the columns indicated by distinctive color groups: severity class of the compound, time points, and dose level. These variables describe the exposure condition applied to the cells. Each row of the heatmap shows the log2 FC values of each gene from every sample where red color shows upregulation and blue color shows downregulation. The column clustering of the heatmap is performed using the “*Ward d2*” algorithm applied to the Euclidian distance between aggregated variables (mean of the log2 FC values from all the dose levels and time points per compound). The row clustering of the heatmap is performed using the “*Ward d2*” algorithm applied to the Euclidian distance between genes. E, A heatmap containing information of modulation (log2 FC) of the selected genes upon the exposure of DILI with various severity levels and non-DILI compounds. The heatmap contains 4 variables on the columns indicated by distinctive color groups: severity class of the compound, categorical class of the compounds, time points, and dose level. These variables describe the exposure condition applied to the cells. Each row of the heatmap shows the log2 FC values of each gene from every sample where red color shows upregulation and blue color shows downregulation. The column clustering of the heatmap is performed using the “*Ward d2*” algorithm applied to the Euclidian distance between aggregated variables (mean of the log2 FC values from all the dose levels and time points per compound). The row clustering of the heatmap is performed using the “*Ward d2*” algorithm applied to the Euclidian distance between genes.

We reasoned that the module associations with cell death could be indicative for causal involvement. Therefore, the causality of the high correlated modules was further evaluated experimentally using RNA interference-based gene silencing of the gene members of the high correlated modules that showed strongest perturbations by DILI compounds (log2 FC > 2 at least in one condition and adj *p* < .1). From total 141 genes (Supplementary Table 6), we selected 67 gene targets to be perturbed based on availability in a druggable siRNA library, and, hence, suspected involvement in cell signaling programs (Supplementary Table 7). Our knock-down protocols resulted in high efficiency based on the lower expression of CHOP-GFP upon tunicamycin exposure in perturbed cells (siDDIT3) compared with siNo1 (Supplementary Figure 7A). After siRNA knock down, HepG2 cells were exposed to nitrofurantoin and nefazodone, 2 DILI compounds that showed strongest cell death onset and transcriptomic perturbation. We anticipated that genes involved in protective adaptive responses would enhance cell death upon knock down, whereas genes that stimulate the onset of cell death would be protective upon knock down. The *z*-score analysis showed that knock down of some candidate genes reduced both apoptotic and necrotic cell death at 24 h after the exposure with nefazodone and nitrofurantoin, whereas knock down of other genes enhanced cell death (Supplementary Table 8). The candidate genes clustered into a cytotoxicity protective group after knock down, including *GTPBP2*, *HSPA1B*, *TSC22D3*, *SIRT1*, *IRF1*, and another 34 genes in the blue box as well as a cytotoxicity enhancing group including amongst others *DDIT3*, *MARCH6*, *SLC6A9*, *SLU7*, *HBP1, MYCL1* in the red box (Figure 5B). Interestingly, we found that the knock down of proapoptotic protein *DDIT3* increased the cell death values upon the cell injury. Previous studies also reported the increase of the cell death upon the perturbation of DDIT3 expression (Wijaya *et al.*, 2021; Yang *et al.*, 2020) suggesting different roles of this protein during cellular stress. We further focused on the target genes that reduced the cell death upon knock down for which cells persistently showed the decrease of cell death onset up to 72 h of compound treatment. Cytotoxicity by nitrofurantoin was likely protected by GTPBP2 for prolonged time period. Nefazodone-induced cell death was most strongly protected by siGFPBP2, siIRF1, and siSIRT1 up to 72 h. In conclusion, we discovered genes that are part of modules associated with cell death onset that are causally related to cell death onset or adaptive response, thus supporting the relevance of the statistical module activity associations with cytotoxic outcomes. Finally, we wanted to ensure that our selected genes were also modulated in PHH exposed to DILI compounds. We used the TG-GATEs dataset (Callegaro *et al.*, 2021) and identified 37 out of the 39 genes (HSP90AA1 and NGFR are not included in the PHH data) whose perturbation reduced cell death and evaluated their expression in PHH after DILI compound treatment (Supplementary Figure 7B). Five genes that showed most significant protection against cytotoxicity after siRNA treatment—*GTPBP2*-HepG2:38, *HSPA1B*-HepG2:64, *IRF1*-HepG2:41, *SIRT1*-HepG2:31, and *TSC22D3*-HepG2:235—exhibited prominent upregulation upon exposure with DILI compounds in PHH (Figure 5D). Moreover, modulation of these genes distinguished the activity between (high severity) DILI compounds with (low severity) non-DILI compounds suggesting the specificity of these genes towards liver injury (Figure 5E).

## Discussion

In this current study, we applied network-based analysis on a large novel concentration and time response transcriptomic dataset of HepG2 cells exposed to more than 40 substances including 18 DILI compounds, 3 cytokines, 7 growth factors, 7 cellular stress responses pathway reference compounds, and 6 non-DILI compounds. Based on this transcriptomics dataset, we successfully established HepG2 cell-specific WGCNA-based gene networks encompassing the spectrum of cellular biological responses and defined the preservation of gene regulation between HepG2 and PHH. Key gene networks were identified that are associated with onset of cell death and candidate representatives from these networks were involved in modulation of cell death. These genes were also modulated by various DILI compounds in PHH, with most considerable changes observed for *HSPA1B*, *TSC22D3*, *GTPBP2*, and *SIRT1*.

We established HepG2 gene networks to facilitate the interpretation of toxicogenomic datasets by reducing the data dimensionality, the ability to assign cellular function and responses for each network, and to quantify the biological activation of the gene networks. This network-based toxicogenomic landscape was also able to distinguish between (high severity) DILI compounds and (low severity) non-DILI compounds according to the differences in module activity. Moreover, our module-based approach captured the gene networks related to various cellular responses linked to the mechanisms of DILI such as stress responses (HepG2:46, HepG2:33) (Wijaya *et al.*, 2021; Wink *et al.*, 2014), ER stress (HepG2:38, HepG2:64, HepG2:65) (Liu and Green, 2019), mitochondrial damage (HepG2:70, HepG2:282) (Ramachandran *et al.*, 2018), and inflammation (HepG2:75, HepG2:140) (Woolbright and Jaeschke, 2018). Complementary, we identified the repressed cellular responses such as organelle biogenesis (HepG2: 24, HepG2: 37), small-molecule metabolism activity (HepG2: 58, HepG2: 69, HepG2: 251), and cell cycle activation (HepG2: 10, HepG2: 252) known to play a role in liver regeneration (Dewhurst *et al.*, 2020; Huang and Rudnick, 2014; Xu *et al.*, 2021). Importantly, preservation analysis showed that 30% of HepG2 modules are preserved in PHH. In particular, the ER-related modules and to a slightly lesser extent also modules related to other cellular injury response pathways, are preserved in PHH. This suggests that, in general, the transcriptional programs that are driving cellular stress response pathways are maintained between primary liver hepatocytes and more dedifferentiated models such as HepG2. Of interest, the cellular stress response gene networks are also well-preserved across species (human vs rat) (Callegaro *et al.*, 2021). The robust intersystem species preservation of the adaptive stress responses could be exploited to improve the translatability across test systems. The overall moderate preservation between HepG2 and PHH likely relates to the fact that HepG2 cells are transformed, dedifferentiated, and highly proliferative, requiring different wiring of transcriptional programs to drive HepG2 biology. However, because the PHH WGCNA was based on a larger number of substances, we cannot exclude that further refinement of the gene network organization could be achieved when more substances that impact of the biology of HepG2 cells would have been included. Regardless, we were able to define the conserved mechanisms between HepG2 and PHH, thereby delineating the biological applicability domain of the HepG2 test system to be used in a low tier test system approach that considers the HepG2 advantages in terms of cost, time, availability, and robustness.

We have correlated HepG2 module EGs scores with cell death outcomes (Wijaya *et al.*, 2021) across the entire compound set, aiming to find common cellular responses leading to cellular adverse outcomes. This allowed us to identify the cellular responses that are activated to initiate repair and/or provide resilience to cell injury (adaptive mechanisms) and that are causally contributing to the adverse cell biological outcomes (adverse mechanisms). We revealed 11 modules with high positive correlation toward apoptotic and/or necrotic cell death of HepG2. A unique avenue in our current work has been to validate the association of these modules with cellular adverse outcomes by assessing the modulation of the cell death after nefazodone and nitrofurantoin treatment using RNA interference of selected genes. We sought a proof-of-concept and selected 67 genes that are contributing to cell signaling based on availability in a druggable siRNA genome library. We found 5 target genes that strongly reduced DILI compound-induced cell death upon the silencing *GTPBP2*, *TSC22D3*, *SIRT1*, *IRF1*, and *HSPA1B*. These results were indicative of the likely causal relationship between their upregulation and cell death. Of interest, these 5 genes are also modulated in PHH by various DILI compounds. Our RNA interference studies were based on individual gene depletion, thereby representing only a minor portion of the gene network. Thus, the causal contribution of the entire gene network is likely underestimated. One could foresee combined depletion experiments of multiple target genes or modulate the activity of transcription factors that are upstream of the individual gene network. Alternatively, CRISPR-Cas9 pooled library screens as they are applied to uncover resistance mechanisms against anticancer drugs, may open additional opportunities to more broadly map the causal-related genes of critical gene networks. Ultimately, with higher throughput of gene perturbation approaches, more DILI compounds could be incorporated to statistically improve the selection of the genes increasing the confidence of the outcomes.

We further highlight the possible mechanisms of the 5 identified potential transcriptional key events that modulate DILI cell death. GTPBP2 is a family of GTP-binding proteins that have GTP hydrolase activity, and play an important role in cell signal transmission, cytoskeletal regulation, protein synthesis, and other activities (Jie *et al.*, 2021). Although the mechanisms of GTPBP2 related to the hepatocellular injury is yet to be discovered, multiple studies have reported the involvement of GTPBP2 in ER stress and by interacting with ATF6 reducing its activity (Jaberi *et al.*, 2015; Kalathur *et al.*, 2012). The involvement of GTPBP2 in the ER stress is reflected by its parent module (HepG2:38) for ER stress-related responses. TSC22D3 or glucocorticoid-induced leucine zipper is associated with the glucocorticoid sensitivity also involved in the inflammation programs (Kalathur *et al.*, 2012). The upregulation of TSC22D3 was reported to be responsible for the cell death in cancerous cells possibly due to the activity of TSC22D3 to inhibit cell proliferation of the cancer cells (Kervoëlen *et al.*, 2015). Thereby, the upregulation of *TSC22D3* might reduce the capacity of the cells to progress through the cell cycle and contribute to tissue regeneration. A previous study has attributed the effect of glucocorticoid on the activity of adenylate cyclase (Aksoy *et al.*, 2002). Specifically, the TSC22 protein family showed direct binding with this enzyme (Kamimura *et al.*, 2021). Moreover, the fact that *TSC22D3* is in WGNCA|HepG2:235 annotated for “adenylate cyclase-inhibiting activity” suggested the connection between activity of this enzyme with proliferation. SIRT1 (sirtuin1) is a member of class III histone deacetylase as a part of HepG2:31 annotated for “nuclear part.” The upregulation of SIRT1 in cells is found to be concurrent with NF-κB inflammation pathway-induced cell death (Chao *et al.*, 2017). IRF1 (interferon regulatory factor-1), a member of HepG2:41 annotated for “transcription factor”; IRF1 is a transcription factor regulating the gene expression during inflammation (Eckhardt *et al.*, 2014). IRF1 has been reported to mediate liver damage during ischemia-reperfusion injury by activating the immune responses during the ischemic episodes (Yan *et al.*, 2020). HSPA1B is a member of heat shock protein 70 family and might play a role in the mechanisms of cell death related to DILI. *HSPA1B* is also a gene member of WGNCA|HepG2:64 which is annotated for “responses to heat.” Although heat shock protein families are known to repair the damaged cells (Yu *et al.*, 2021) (also showed by our results in Figure 5B—increase of cell death upon the perturbation of *HSPB1* and *HSPA8*), the upregulation of HSPA1B has been previously linked to the inflammation-induced cell death (Calderwood *et al.*, 2012; Daniels *et al.*, 2004). Altogether, we managed to establish these novel transcriptomics key events. Despite these findings, further work needs to be performed to fully verify the detailed mechanism of these genes in the course of DILI.

In conclusion, we have applied WGCNA to novel TempO-Seq toxicogenomic dataset. We anticipate that test system-specific gene network-based approaches are powerful to efficiently mine the biology from toxicogenomic studies and contextualize the biology of the individual test systems through preservation statistics. Similar approaches would be worthwhile for other liver test systems that are relevant for DILI prediction, including HepaRG cells, iPSC-derived hepatocyte-like cells, and liver microtissues. This would contribute to an improved understanding of the fit for purpose of individual liver test systems for mechanism-based safety assessment.

## Supplementary Material

kfad121_Supplementary_Data

## Data Availability

The transcriptomic data have been made available on https://www.ebi.ac.uk/fg/annotare/ with accession number E-MTAB-11555 for the first transcriptomic batch and E-MTAB-11578 for the second transcriptomic batch. These datasets are still private thereby having limited accessibility. If there is a need to access the data, the permission will be given upon request. The imaging data will be also given upon request. The data will be made publicly accessible upon the publication of the manuscript.
