# Supplementary material for: A network-based transcriptomic landscape of HepG2 cells uncovering causal gene-cytotoxicity interactions underlying drug-induced liver injury
Source: Toxicol Sci. 2023 Nov 28;198(1):14–30. doi: 10.1093/toxsci/kfad121 (PMC10901150; doi:10.1093/toxsci/kfad121)
Supplement: kfad121_Supplementary_Data [file kfad121_supplementary_data.zip › kfad121_Supplementary_Data/toxsci-23-0257-File007.docx]

**A Network-based Transcriptomic Landscape of HepG2 cells Uncovering Causal Gene-Cytotoxicity Interactions Underlying Drug-Induced Liver Injury**

Lukas S. Wijaya^1^, Attila Gabor^2^, Iris E. Pot^1^, Luca van de Have^1^, Julio Saez-Rodriguez^2^, James L. Stevens^1^, Sylvia E. Le Dévédec^1^, Giulia Callegaro^1^, Bob van de Water^1#^

^1^Leiden Academic Centre for Drug Research (LACDR), Leiden University, Leiden, The Netherlands

^2^Heidelberg University, Faculty of Medicine, and Heidelberg University Hospital. Institute for Computational Biomedicine, 69120 Heidelberg, Germany

^#^ Corresponding author: Bob van de Water (b.water@lacdr.leidenuniv.nl), Leiden Academic Centre for Drug Research, Leiden University, Einsteinweg 55, 2333 CC Leiden, The Netherlands

Supplementary Figures


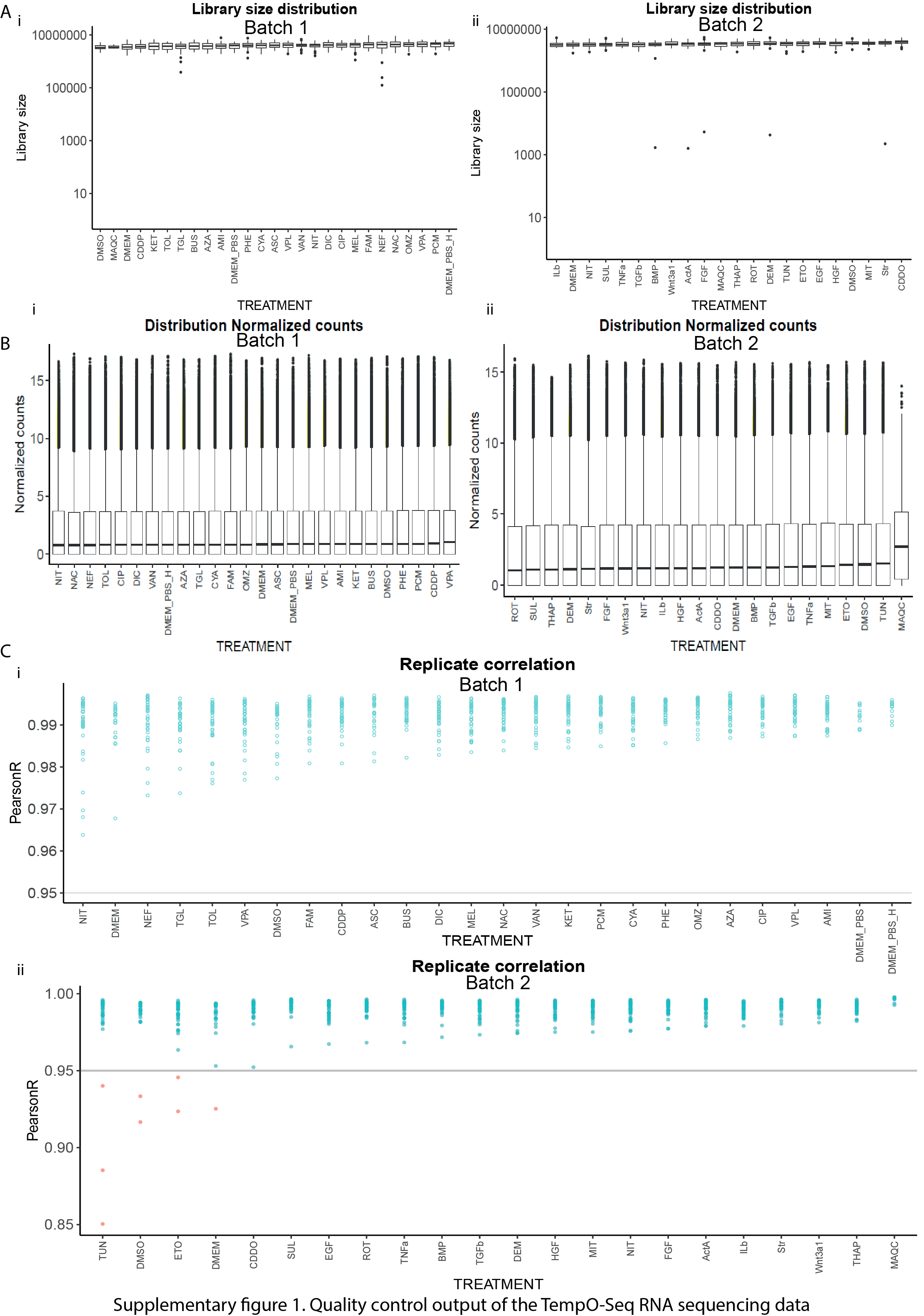


**Supplementary figure 1. Quality control output of the TempO-Seq RNA sequencing data.** (A) the library size of the samples grouped per compound from batch 1 (i) and batch 2 (ii). Each dot represent one samples. (B) The distribution of the CPM normalized count from batch 1 (i) and batch 2 (ii) grouped per compound. Each dot represent the read from one probe. (C) The correlation value (Pearson correlation) based on the CPM normalized count from batch 1 (i) and batch 2 (ii) of each samples to the mean value derived from the samples exposed to the same conditions (replicate). Each dot represent the correlation value from each sample. The color of the dots indicate the samples passing the threshold (Pearson correlation > 0.95 – blue : pass, red : not-pass).


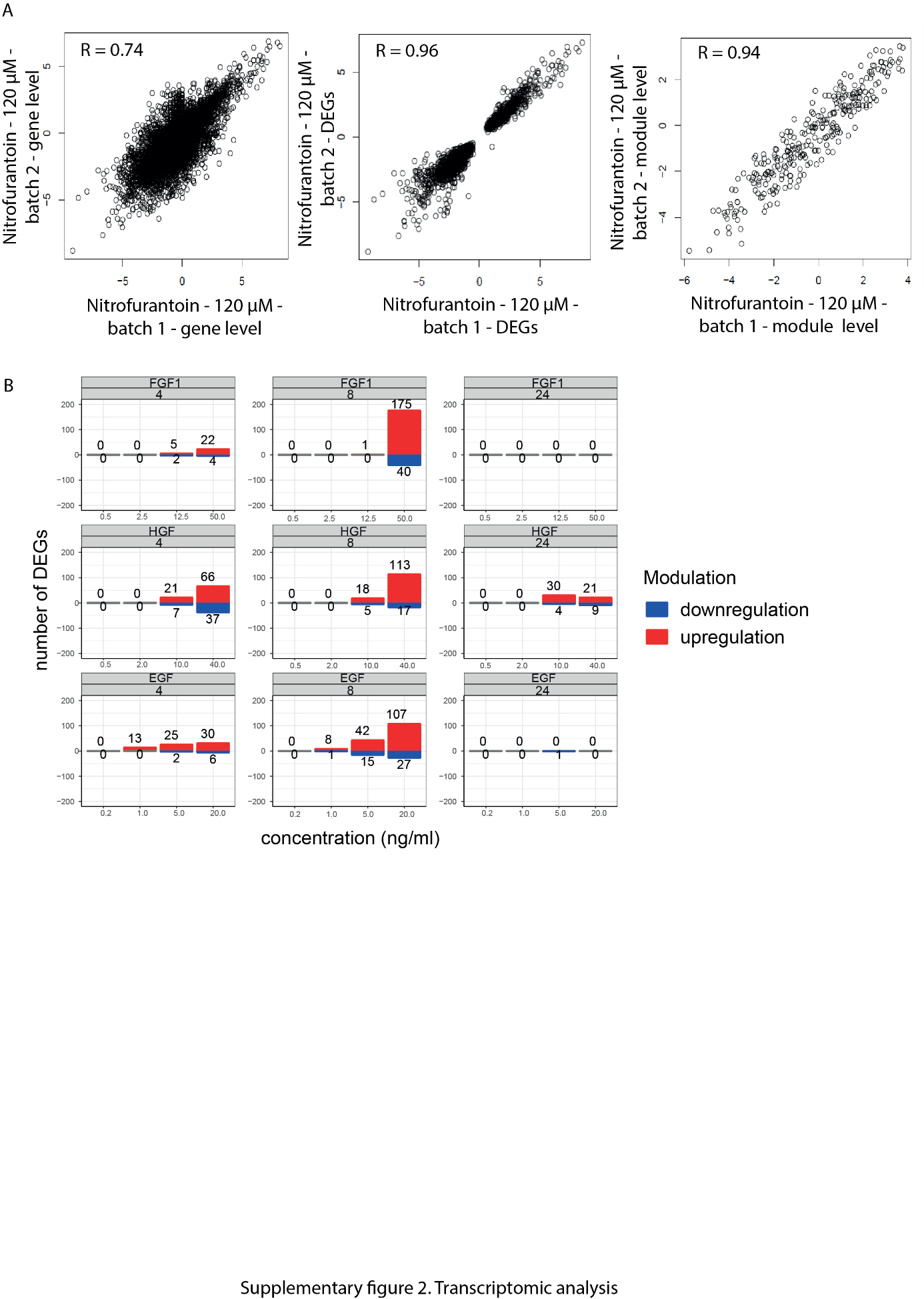


**Supplementary figure 2. Transcriptomic analysis.** (A) The plot showing differential expressed genes number of FGF, HGF, and EGF at 8 hours (top) and 24 hours (bottom). Red bars indicate upregulated genes and blue bars indicate downregulated genes. (B) The correlation plot derived from the transcriptomic responses at the gene level (left), DEGs level (middle), and module responses (right) between nitrofurantoin (120 µM – 24 hours) from the batch 2 (y-axis) vs nitrofurantoin batch 1 (x-axis). Each dot shows the log2 fold change value of each gene (left) or module (right). The threshold of the DEGs is set with adjusted p-value < 0.01 and log2 fold change > [0.1]


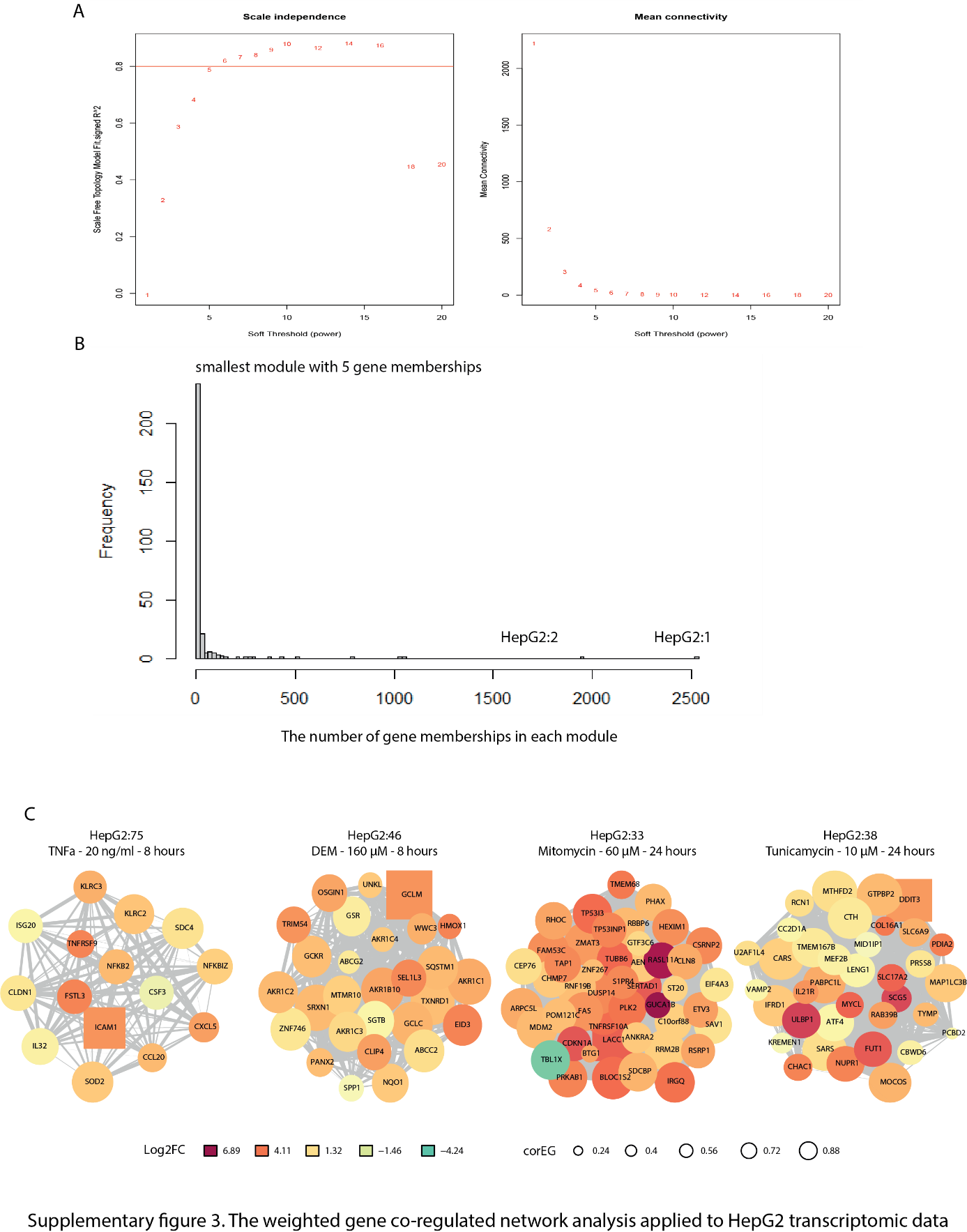


**Supplementary figure 3. The weighted gene co-regulated network analysis applied to HepG2 transcriptomic data.** (A) The plot showing the pattern of scale free topology versus soft threshold power (left) and mean connectivity versus soft threshold (right). (B) The distribution of the size of the modules derived from the HepG2 transcriptomic data.


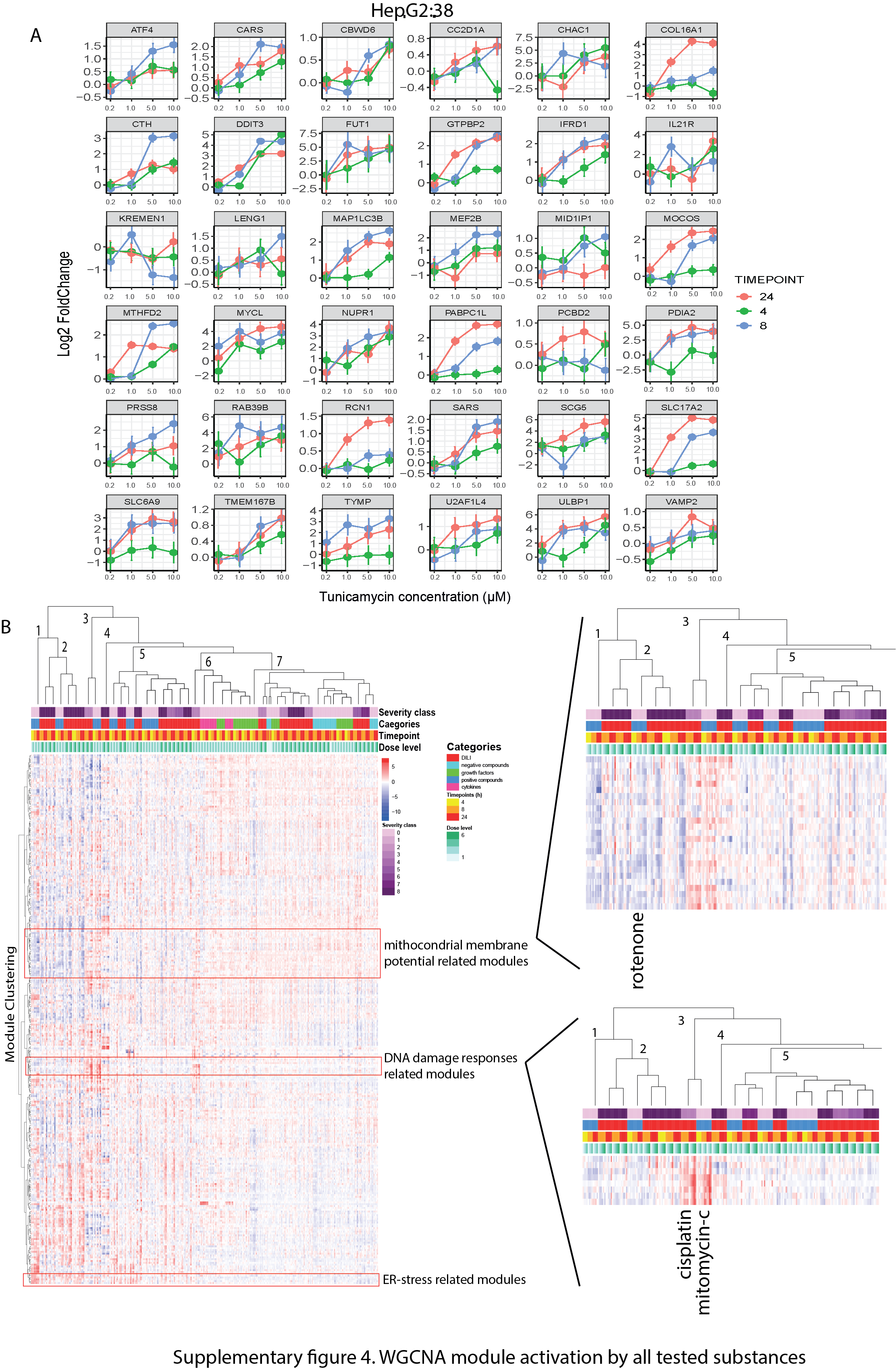


**Supplementary figure 4. WGCNA module activation by all tested substances.** (A) The modulation of the gene memberships of HepG2:38. The color of the plots represents the time point, the error bars indicate the standard error mean (SEM). (B) Heatmap showing the overview of the module responses. The heatmap contains 4 variables on the columns indicated by distinctive color groups: severity class of the compound, compound categories, time points, and dose level. These variables describe the exposure condition applied to the cells. Each row of the heatmap shows the eigengene scores of each module from every sample where red color shows activation and blue color shows repression. The column clustering of the heatmap is performed using the “*Ward d2*” algorithm applied to the Euclidian distance between aggregated variables (mean of the eigengene scores from all the dose levels and time points per compound). The row clustering of the heatmap is performed using the “*Ward d2*” algorithm applied to the Euclidian distance between modules. The red boxes highlighted module clusters of mitochondrial membrane potential, DNA damage responses (enlarged in the inset), and ER-stress related modules (further explained in Figure 2D).


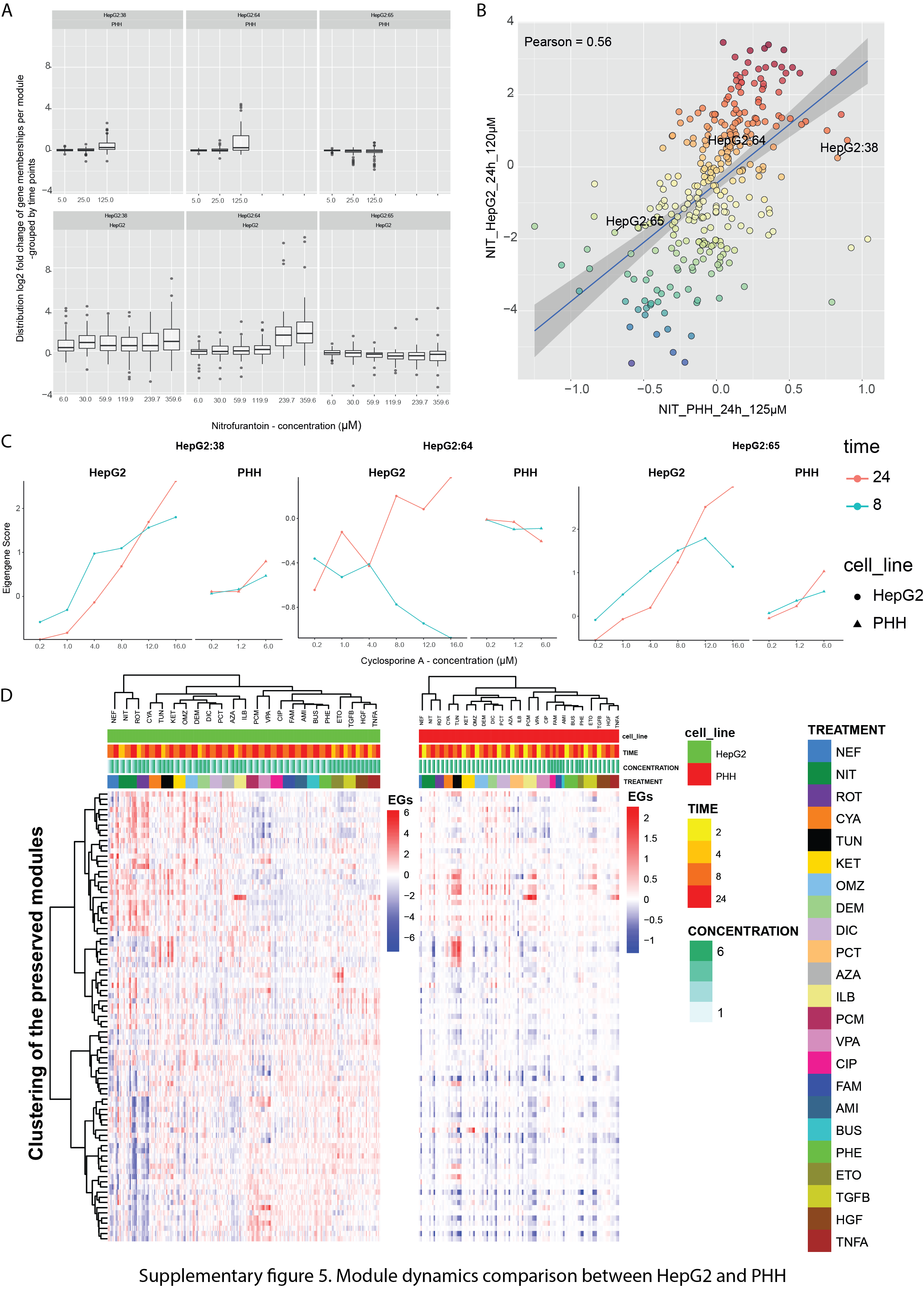


**Supplementary figure 5. Module dynamics comparison between HepG2 and PHH.** (A) Distribution plots of log2 fold change values of gene memberships of HepG2:38, HepG2:64, and HepG2:65 grouped by time points in HepG2 and PHH upon the exposure of nitrofurantoin. (B) Module correlation plot between PHH and HepG2 exposed to nitrofurantoin (125 µM and 120 µM respectively) for 24 hours. Each dot on the correlation represents each module activity. The ER stress related modules are highlighted : HepG2:38, HepG2:64, HepG2:65. (C) Response of HepG2 and PHH upon cyclosporine A exposure based on the HepG2:38, HepG2:64, and HepG2:65. The colors of the plot represent time points and the shapes of the dots indicate the cell lines. (D) Heatmap displaying module activity based on the preserved modules. The heatmap shows 4 different variables : cell line, time, concentration, and treatment. Left panel HepG2 TempO-Seq and right panel PHH TG-GATEs Affymetrix arrays. The colors of the heatmap indicate the direction of the modulation (red : activation, blue : deactivation). Each line of the heatmap displays the activity of each module according to the indicated variables.


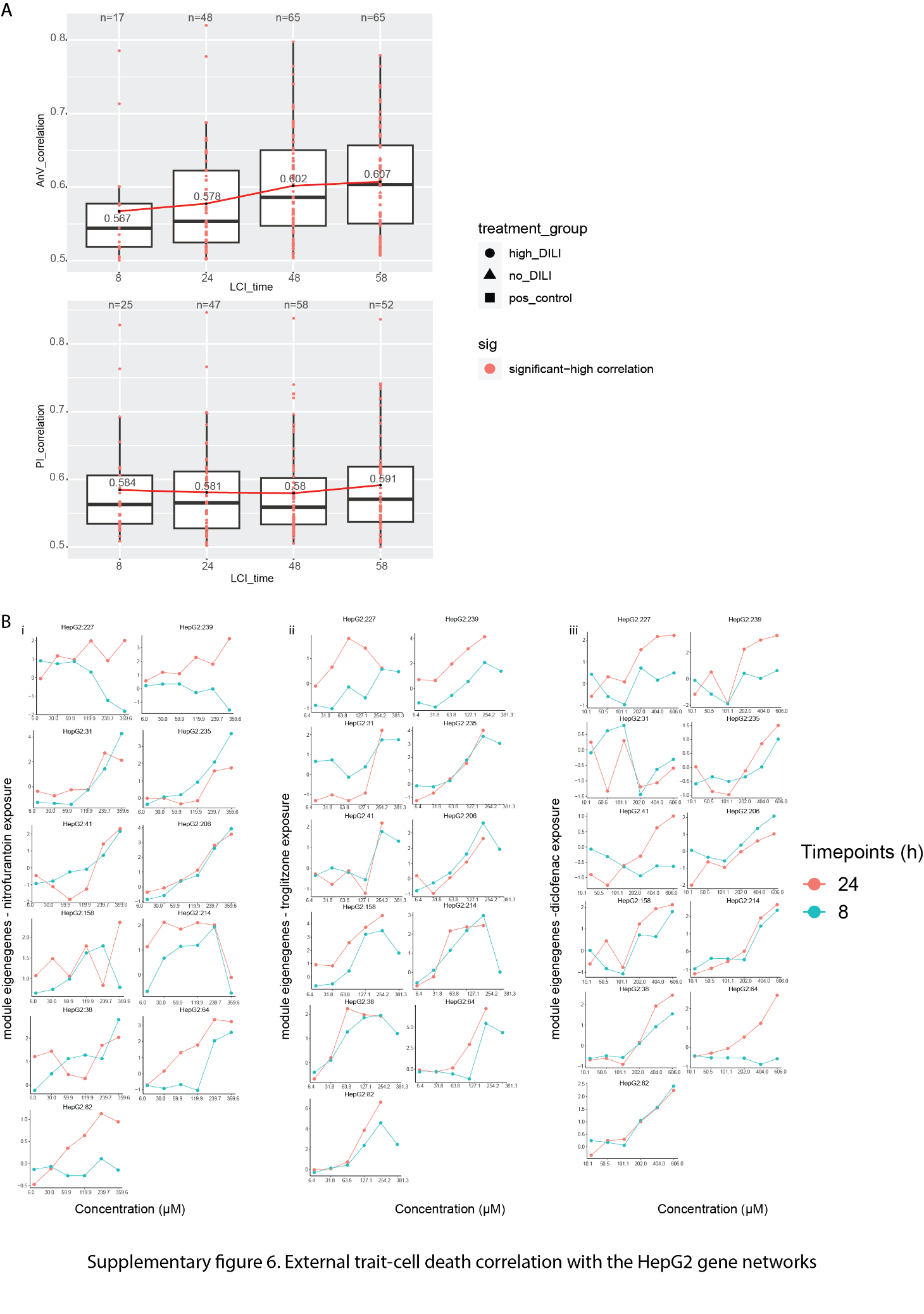


**Supplementary figure 6. External trait-cell death correlation with the HepG2 gene networks.** (A) Distribution plots of significant-high correlated pairs of the module responses of every tested compound for each module towards cell death measured with annexin V (top) and PI (bottom) in different time points of cell death measurement (8, 24, 48, 58 hours) (threshold : p-value < 0.1 , Pearson correlation score > 0.5). Every dot represents the correlation value between module responses and cell death measurement of each tested compound. Shapes of the points display group of the compounds. (B) The plots showing of the dynamics of 11 highest cell death-correlated modules upon the exposure of nitrofurantoin (i), troglitazone (ii), and diclofenac (iii). The colors of the plots represent time point.


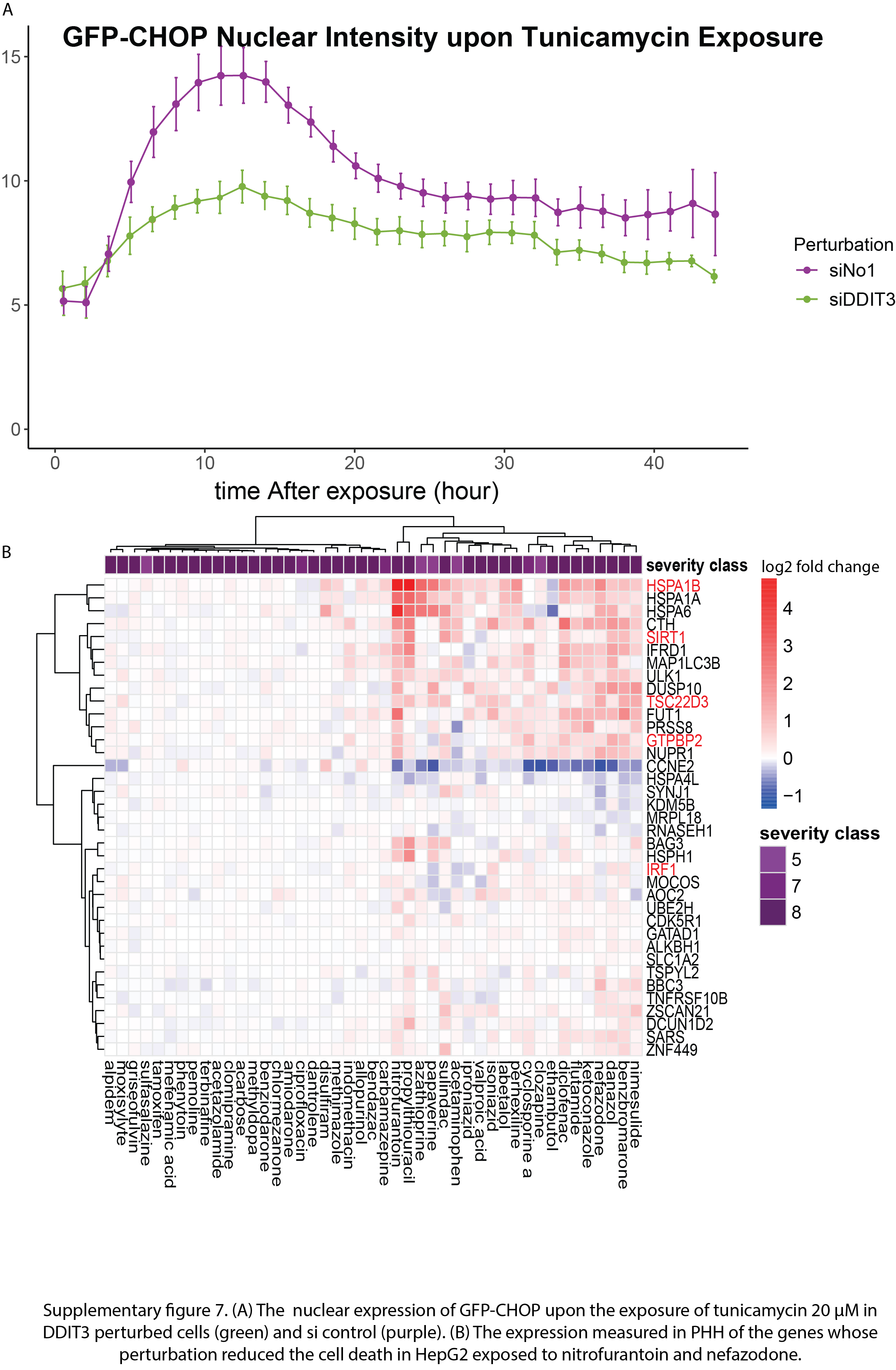


**Supplementary figure 7.Additional information supporting the outcomes of the RNA interference experiment.** (A) Temporal dynamics of nuclear expression of CHOP-GFP upon the exposure of 60 µM of tunicamycin in cells with siDDIT3 (green) and siNo1-control (purple) measured for 48 hours. The measurement was performed every 1.5 hours. The values were the average of 4 different images with the standard deviation value represented as error bars. (B) The heatmap shows the log2 fold changes of 18 out of 20 (2 genes are not measured in the TG-GATEs (Barabási et al. 2011)) in PHH of the genes whose perturbation decreased the cell death fraction in HepG2 upon the exposure of nitrofurantoin (360 µM) and nefazodone (39.5 µM). The heatmap contains 3 variables on the columns indicated by distinctive color groups: severity class of the compound, time points, and dose level. These variables describe the exposure condition applied to the cells. Each row of the heatmap shows the log2 fold change values of each gene from every sample where red color shows upregulation and blue color shows downregulation. The column clustering of the heatmap is performed using the “*Ward d2*” algorithm applied to the Euclidian distance between aggregated variables (mean of the log2 fold change values from all the dose levels and time points per compound). The row clustering of the heatmap is performed using the “*Ward d2*” algorithm applied to the Euclidian distance between genes.
